# Modelling co-development between the somites and neural tube in human trunk-like structures

**DOI:** 10.1038/s41556-025-01813-8

**Published:** 2025-12-16

**Authors:** Komal Makwana, Louise Tilley, Probir Chakravarty, Jamie Thompson, Peter Baillie-Benson, Ignacio Rodriguez-Polo, Naomi Moris

**Affiliations:** 1 The Francis Crick Institute, London, UK; 2 Present Address: The Babraham Institute, Cambridge, UK

**Keywords:** Embryogenesis, Stem cells

## Abstract

Human stem cell-based embryo models provide experimentally amenable in vitro systems for developmental research. A key feature of embryo models is their multi-lineage differentiation, which allows for the study of tissue co-development. Here we develop human trunk-like structures that have morphologically organized somites and a neural tube that form through self-organized, endogenous signalling. Transcriptomic comparison with human embryo datasets suggests that human trunk-like structure cells approximate Carnegie stage 13–14 (28–35 days after fertilization). The absence of a notochord leads to a dorsal identity, but exogenous Sonic Hedgehog signalling activation ventralizes both the somites and the neural tube in a dose-dependent manner. We further identify reciprocal signalling: neural tube-derived cues induce medial ALDH1A2 in somites, which in turn generate retinoic acid signals that drive spontaneous neural-tube patterning. Together, our data highlight the value of modularity in embryo models, which we leverage to explore human trunk co-development.

## Abstract (web summary)

Makwana, Tilley et al. generate human stem cell-based trunk-like structures approximating Carnegie stage 13–14 of development. They use them to model and study the development of the thoracic and lumbar trunk.

## Main

In the embryo, tissues and organs mature alongside neighbouring tissues, using local interactions that are important for correct development and downstream function. For example, neighbouring tissues may provide positional information through spatially localized cues or provide signalling feedback that balances the proportions of cell fates. Inter-tissue interactions are also important for ensuring synchronized developmental timing, so that the embryo keeps a consistent pace. We refer to this inter-dependency between tissues as ‘co-development’.

There is therefore a need to investigate organogenesis at the multi-tissue level. This has been challenging using embryos because signalling pathway convergence and overlapping communication across many tissues can make it difficult to identify reciprocal interactions between any two tissues of interest. Classical experimental embryology provides a route to explore tissue interactions through manipulations including explants, transplants and excisions (see, for instance, refs. ^1–8^). However, these approaches are not feasible in humans owing to ethical and technical constraints, including limited long-term human embryo culture^9^.

Stem cell-based embryo models offer a practical alternative^10^. Their multi-lineage potential enables exploration of inter-tissue interactions, while bottom-up generation of specific tissues in the presence or absence of others can determine the necessity and sufficiency of these interactions^11^. Importantly, human pluripotent stem (PS) cell-derived models provide scalability, experimental flexibility and species-specific relevance that cannot be achieved with embryos alone.

Several models of early embryogenesis have now been developed. Human gastruloids are self-organized aggregates of PS cells that spontaneously break symmetry, differentiate to all three germ layers and undergo axial elongation and spatiotemporal gene expression along an anteroposterior (AP) axis^12^. However, they lack morphological complexity, including somitic epithelialization. Mouse gastruloids can form axially organized morphological somites through embedding in an extracellular matrix gel^13^ and even form integrated somite- and neural tube-containing structures called trunk-like structures (TLSs)^14^. Likewise, three-dimensional (3D) human models of somitogenesis^15–17^, the caudal neural tube^18,19^, structures that contain both neural and somitic mesoderm^20–22^, neuromuscular junctions^23,24^ and notochord progenitors^25^ have been described. These models showcase the remarkable modularity of embryo models to include or exclude particular tissues, in a building block-like manner. We reasoned that this could be harnessed to explore co-development, focusing particularly on the tissue interactions during axial extension.

Somitic and neural tube development needs to be coordinated to form functional organ systems, for instance, the innervation of muscles to form circuits. There is evidence of co-development between these tissues, such that signals from the neural tube promote myogenesis^3,5–7,26^ and prevent medial *Sim1* expression in the somites^2^, while somitic signals to the neural tube promote neurogenesis^8,27^. However, the exploration of these interactions in humans has been hampered by a lack of appropriate experimental systems. Here, we modify a somite-only axioloid protocol to develop robust and reproducible human TLSs (hTLSs) that allow us to explore trunk development in the presence or absence of the neural tube.

We find that, owing to the absence of a notochord, the hTLS exhibits dorsal bias, but exogenous activation of Sonic hedgehog (SHH) signalling induces ventralization in a dose-dependent manner. Notably, even without a localized SHH source, we observed neural tube patterning orthogonal to the anterior–posterior (AP) axis, mediated by somite-derived retinoic acid (RA) signalling, which we validate through an RA responsive element (RARE) reporter system. This RA signalling is required for localized *PAX6* expression in the neural tube and for neurogenesis. Moreover, the neural tube was able to induce *ALDH1A2* expression specifically in medial somites, a finding that we validated in mouse embryos. Our findings highlight the potential of stem cell-based embryo models to uncover the regulatory logic of co-development and show that the modular nature of embryo models can offer insights into the coordination of tissue interactions during early human organogenesis.

## Results

### Establishing an hTLS protocol

**Fig. 1:**
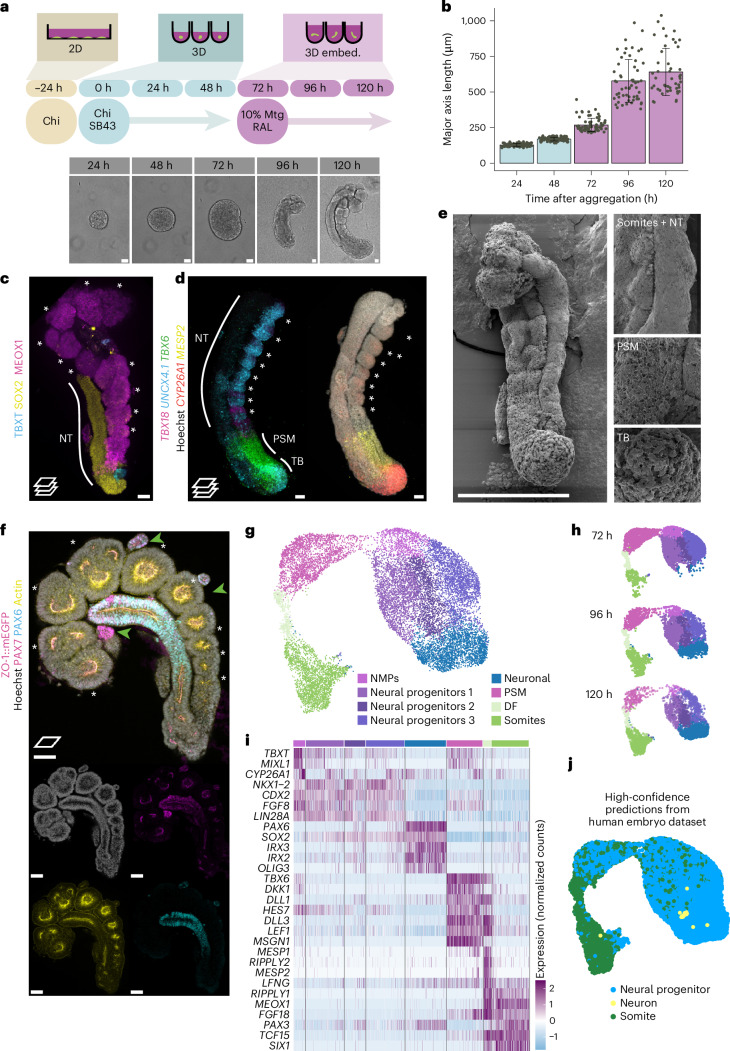
Establishing an hTLS model. **a**, Schematic of the hTLS protocol with representative brightfield images at each 24-h timepoint. 2D, two-dimensional; embed., embedded. **b**, Elongation of ZO-1::mEGFP hTLS major axis length (mean ± s.d.) (*n* = 12–32 per timepoint). **c**, Projection of immunofluorescence imaging of ZO-1::mEGFP hTLS at 120 h (*n* = 6). The asterisk indicates somites. **d**, Projected images of whole-mount, in situ HCR staining of HES7::Achilles hTLS at 120 h (*n* = 2). The asterisk indicates somites. **e**, Scanning electron micrograph of a LaminB1::mTagRFP-T hTLS at 120 h (*n* = 2). Scale bar, 300 μm. **f**, Confocal section (top) and projection (bottom) of immunofluorescence imaging of a ZO-1::mEGFP hTLS at 120 h. Green arrows highlight clusters of PAX7+ cells (*n* = 2). The asterisk indicates somites. Scale bars, 100 μm. **g**, Single-cell transcriptomic UMAP of LaminB1::mTagRFP-T hTLS development (*N* = 1; *n* = 15,808 cells). **h**, The temporal spread of samples collected at 72 h, 96 h and 120 h timepoints. **i**, Marker gene expression for each identified cluster. **j**, Projection and subsequent label transfer of high-confidence cell identities from a published human embryo dataset^38^. Chi, Chiron (CHIR99021); DF, determination front; Mat, Matrigel; NMP, neuromesodermal progenitor; NT, neural tube; PSM, presomitic mesoderm; RAL, retinal; SB43, SB431542; TB, tailbud. Scale bars, 50 μm, unless otherwise stated. Source data

**Extended Data Fig. 1:**
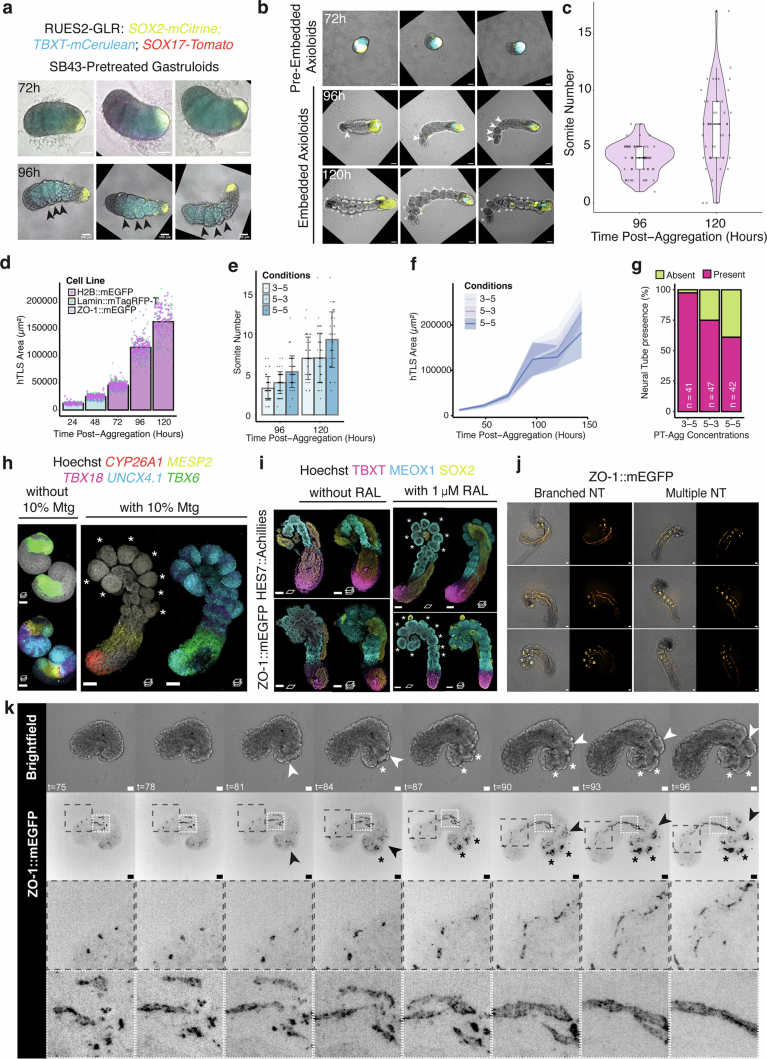
Establishing and characterising the hTLS model. (**a**) Human gastruloids pretreated with the Nodal signalling inhibitor, SB43 (SB431542), show a typical ‘striped’ pattern at 72 h (top), and sequential somite-like structures upon embedding in Matrigel (bottom). Black arrowheads indicate somite-like structure boundaries. (**b**) Morphology of RUES2-GLR axioloids over time. Three representative images are shown. Scale bars, 100 µm. White arrowheads indicate segmenting boundaries, * indicates individual segments. (**c**) Number of somites in ZO-1::mEGFP hTLS over time (mean±standard deviation(sd)) (*n* = 47-55 per timepoint). (**d**) Change in size of ZO-1::GFP, LaminB1::mTagRFP-T, and H2B::mEGFP hTLSs over time (bar indicates mean average). (**e**–**g**) hTLS morphology quantifications following varied exposure to Chi (CHIR99021) at pretreatment (PT) and aggregation (Agg) stages. Cultures were pre-treated with 3 µM Chi, and aggregated with 5 µM Chi (‘3-5’), pre-treated with 5 µM Chi, and aggregated with 3 µM Chi (‘5-3’), or pre-treated with 5 µM Chi, and aggregated with 5 µM Chi (‘5-5’). (**e**) Change in somite number over time (bar indicates mean±standard deviation(sd)). (*n* = 38-55 per condition and per timepoint). (**f**) Change in hTLS size across time (thick line indicates mean, shaded area represents standard deviation(sd). (**g**) Proportion of structures with neural tube present or absent. (**h**) Projected images of 120 h HES7::Achilles hTLS with and without embedding in Matrigel (Mtg). Scale bars, 100 μm. * indicates somites. (**i**) Immunostained optical slice (left) and projections (right) of 120 h ZO-1::mEGFP or HES7::Achilles hTLS with and without exposure to retinal (RAL) from 72-120 h. These hTLS were generated from HES7::Achillies (top) and ZO-1::mEGFP (bottom) cell lines. Scale bars, 100 μm. * indicates somites. (**j**) Representative images of ZO-1:mEGFP hTLSs, showing multiple and branched neural tubes (NT). Scale bars, 50 μm. (**k**) Representative brightfield (top) and confocal images (bottom) of live imaging of ZO-1:mEGFP hTLS at 3 h intervals. White arrows indicate segmenting somite boundaries and * indicates formed somites. Dashed boxes highlight enlarged regions. Scale bars, 50 μm. Source data

While mouse gastruloids embedded in low percentage Matrigel can develop organized epithelial somite structures^13,14^, human gastruloids had to be exposed to the Nodal signalling inhibitor SB431542 (hereafter, SB43) during pre-treatment^12^, to generate segments when embedded in 20% Matrigel (Extended Data Fig. 1a). These segments formed sequentially from the posterior, but they did not form rounded, epithelial structures characteristic of somitic morphology in the embryo. Instead, a protocol combining the Wnt–β-catenin activator CHIR99021 (hereafter, Chi) and SB43 at aggregation with embedding in 10% Matrigel enabled morphologically organized somitic tissue formation as axioloids^15^ (similar to in refs. ^16,17^). We adapted this protocol from induced pluripotent stem (iPS) cells to human embryonic stem (ES) cells (Extended Data Fig. 1b) by adjusting the Chi concentration according to cell line requirements (Methods). In doing so, we noticed that higher concentrations of Chi biased the cultures towards the mesodermal fate, as expected^28^, while lower concentrations enabled the coformation of a morphologically organized neural tube alongside the somites (Fig. 1a). These structures increased in somite number, were elongated and displayed an organized somitic and neural tube morphology in 79% of aggregates (Fig. 1b and Extended Data Fig. 1c–g). Marker gene expression confirmed the presence of somitic (MEOX1+) and neural (SOX2+) identities with a pool of progenitors (SOX2+ and TBXT+) at the posterior and a presomitic mesoderm (PSM) domain (*TBX6*+ and *MESP2*+) in the tailbud region (Fig. 1c,d). Somites expressed *TBX18* anteriorly, particularly in the first one to two newly formed somites, and *UNCX4.1* posteriorly (Fig. 1d). As such, the structures were similar to recently described hTLSs^20–22^, with the important distinction that the protocol could be ‘fine-tuned’ by adjusting the level of Chi to give somite-only or combined somite- and neural tube-containing structures, thereby allowing direct comparison between structures with the presence or absence of the neural tube.

**Extended Data Fig. 2:**
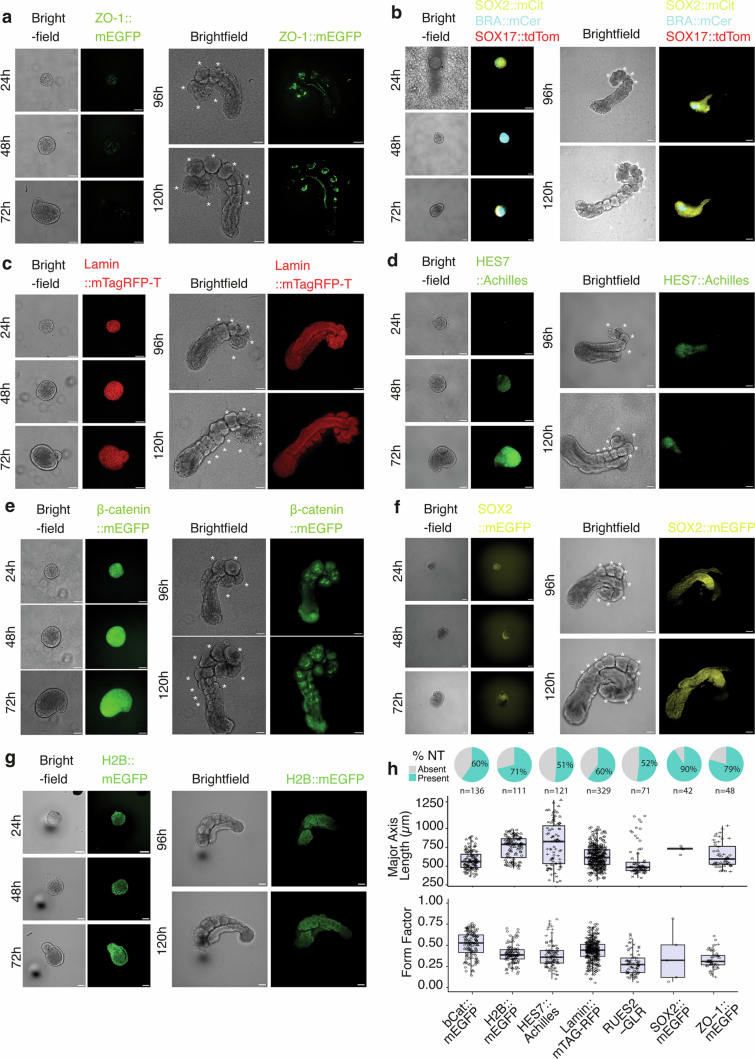
Generation of hTLS from several cell lines. (**a**–**f**) Representative brightfield images of hTLS across timepoints, alongside fluorescent reporter expression for (**a**) ZO-1::mEGFP (n = 48), (**b**) RUES2-GLR (n = 71), (**c**) LaminB1::mTagRFP-T (n = 329), (**d**) HES7::Achilles (n = 121), (**e**) β-catenin::mEGFP (n = 136), (**f**) SOX2::mEGFP (n = 42) and (**g**) H2B::mEGFP (n = 111). (**h**) Quantification of the presence of a neural tube (NT; top), major axis length (middle) and form factor (bottom) by cell line (*n* = 42-329 per cell line). Scale bars, 100 μm. Source data

Similar to in other in vitro models of somitogenesis^15,21^, we noted that the morphological organization of the hTLS was critically dependent on embedding in Matrigel and exposure to retinal, as time-matched structures without these had some degree of gene expression organization along an AP axis but no or little morphological organization of somites or the neural tube (Extended Data Fig. 1h,i). We were also able to adapt the protocol to a total of ten cell lines (from five parental backgrounds), including both human ES and iPS cells, which showcases the versatility of the protocol beyond intrinsic line-to-line variability (Methods; Extended Data Fig. 2). Detailed step-by-step instructions for forming hTLSs are available at 10.17504/protocols.io.e6nvw4d59lmk/v1 (ref. ^29^).

### Characterizing hTLS formation

To better understand the morphological organization of hTLSs, we performed scanning electron microscopy (SEM) to visualize the surface morphology. This showed cell compaction differences, with loosely arranged cells in the posterior tailbud, medium compaction in the PSM and tightly compacted epithelialization in both the neural tube and somite domains (Fig. 1e), in keeping with descriptions in vertebrate embryos^30,31^. Likewise, the hTLS generated with a ZO-1::mEGFP line showed that both the somites and neural tube had internal ZO-1- and actin-rich lumina, with cells organized as epithelia (Fig. 1f). We therefore wanted to better understand how these epithelial tissues formed in the hTLS.

This question is particularly interesting for the neural tube because it is known to form through two modes of morphogenesis in mammals: primary and secondary. Primary neurulation involves the folding of the neural ectoderm^32^ and has been extensively studied because malformations are the leading cause of neural tube defects (NTDs) in humans^33^. By contrast, secondary neurulation—when neural cells condense to form rods that later epithelialize to form a contiguous tube—is much less understood because it accounts for only the caudal-most portion of the axis^34^. Exactly how secondary neurulation occurs in humans, how the two modes are coordinated and whether the human embryonic neural tube is branched at early stages or contiguous^35^ are still open questions in the field. Using the ZO-1::mEGFP cell line, we sometimes saw multiple or branched neural tubes, particularly at the posterior pole (Extended Data Fig. 1j). With live imaging, we observed epithelial cysts that progressively merged to generate a contiguous tract (Extended Data Fig. 1k and Supplementary Video 1). Therefore, it seems that the mode of neural tube formation in hTLS structures is similar to secondary neurulation and may prove useful in establishing the cell biological mechanisms of such phenomena in vitro.

**Extended Data Fig. 3:**
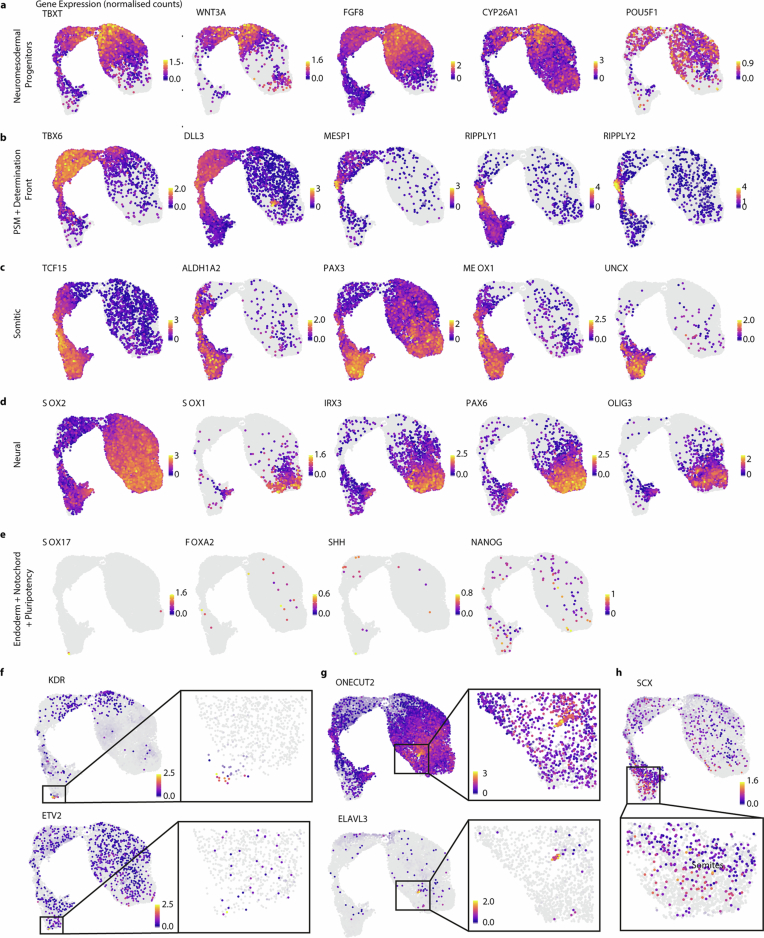
Gene expression signature of hTLS single cells. Cells in UMAP space coloured by gene expression for markers of neuromesodermal progenitors (**a**), the presomitic mesoderm (PSM) and determination front (**b**), somitic mesoderm (**c**), neural tissue (**d**), and markers of endoderm (*SOX17, FOXA2*), notochord (*SHH*) and pluripotency (*NANOG*; **e**). Zoomed-in regions are also shown for small sub-populations of cells expressing endothelial markers (*KDR*, *ETV2*; **f**), neural differentiation markers (*ONECUT2*, *ELAVL3*; **g**) and sclerotome (*SCX*, **h**). Source data

**Extended Data Fig. 4:**
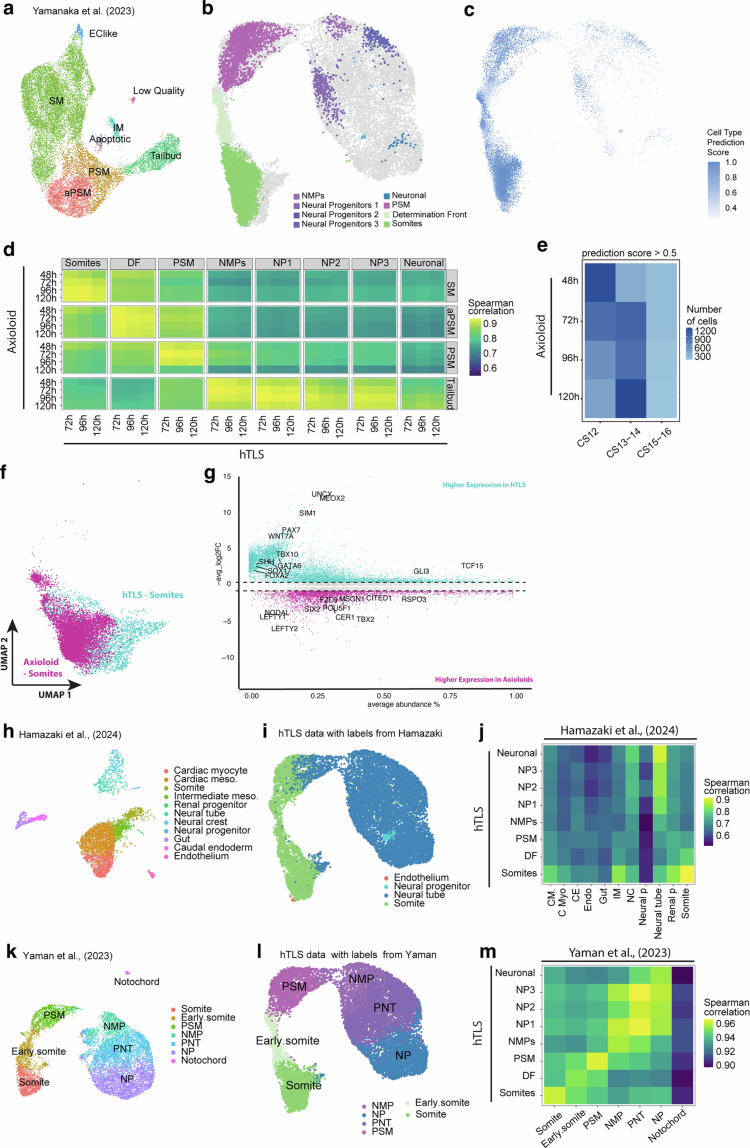
Comparison between hTLS and an axioloid dataset. (**a**) UMAP projection of an axioloid dataset^1^, with clusters annotated according to published labels. (**b**) Projection of axioloid single cells onto the hTLS UMAP, coloured by label transfer from hTLS cluster identity. Note that the somitic lineages are well populated by axioloid cells, while the neural identies are mostly absent. (**c**) Projected cell coloured by prediction score as a measure of confidence. (**d**) Correlation in gene expression between timepoints in the axioloid and hTLS datasets, by cell cluster. (**e**) Label transfer of axioloid cells onto an embryonic dataset^2^ showing maximal number of cells at the CS13-14 stage. (**f**) Comparison between axioloid somitic mesoderm (SM) and hTLS somite clusters. Cells within the overlapping region were taken for downstream processing including differential gene expression analysis. (**g**) MA plot for differentially expressed genes bewteen the axioloid and hTLS somitic clusters. Some significantly different genes of interest are annotated. (**h**–**m**) Analysis of alternative somite- and neural tube-containing in vitro models compared to hTLS data shown here. (**h**–**j**) Single cell data from Hamazaki and colleagues^3^, or (**k**–**m**) Yaman and colleagues^4^, was plotted by UMAP using the original annotations (**h**, **k**), and then projected onto the hTLS dataset for label transfer (**i**, **l**). Additionally, the data was compared by Spearmans Correlation to the hTLS populations (**j**, **m**). IM, Intermediate mesoderm; EC, Endothelial Cells; SM, Somitic mesoderm; PSM, Presomitic mesoderm; aPSM, anterior PSM; DF, Determination Front; NMPs, Neuromesodermal progenitors; NP, neural progenitor; MG, Matrigel; RAL, Retinal; PNT, Posterior Neural Tube; CS, Carnegie Stage. Source data

To further characterize hTLSs over time, we collected samples at 72 h, 96 h and 120 h and performed 10× single-cell RNA sequencing. We detected populations including a neuromesodermal progenitor (NMP) cell state (expressing *SOX2* and *TBXT*), PSM (*TBX6*, *HES7* and *DLL1*), determination front (*RIPPLY1/2* and *MESP1/2*), somitic (*MEOX1* and *TCF15*) and neural (*SOX2* and *PAX6*) clusters (Fig. 1g and Extended Data Fig. 3a–e). Cells from early timepoints mostly populated the progenitor populations, while those from later timepoints populated more mature states (Fig. 1h). In addition, we detected sub-clusters including endothelial cells (*KDR*+ and *ETV2*+; Extended Data Fig. 3f) that match previous reports of a somite-derived endothelial population in mouse TLSs^14^, human axioloids^15^ and embryos^36,37^. In addition, we observed a small population of mature neural cells (*ONECUT2* and *ELAVL3*; Extended Data Fig. 3g), indicating that neurogenesis starts at 120 h in the hTLS. By contrast, we saw no evidence of notochord, non-neural ectoderm, neural crest, endoderm or lateral plate mesoderm fates, corroborating the observation that the hTLSs contain only neural tube and somitic progenitors (Fig. 1i). Bioinformatic projection onto an existing axioloid dataset^15^ confirmed that the hTLSs contain neural cell types not present in the somite-only version and also showed some differences in terms of gene expression in the two overlapping somitic clusters, indicating that, while comparable, hTLSs are indeed distinct entities from axioloids (Extended Data Fig. 4a–g). The hTLSs also showed similarities with other somite- and neural tube-containing models^21,22^ but with some notable differences, such as the absence of a notochord or endoderm (Extended Data Fig. 4h–m).

**Extended Data Fig. 5:**
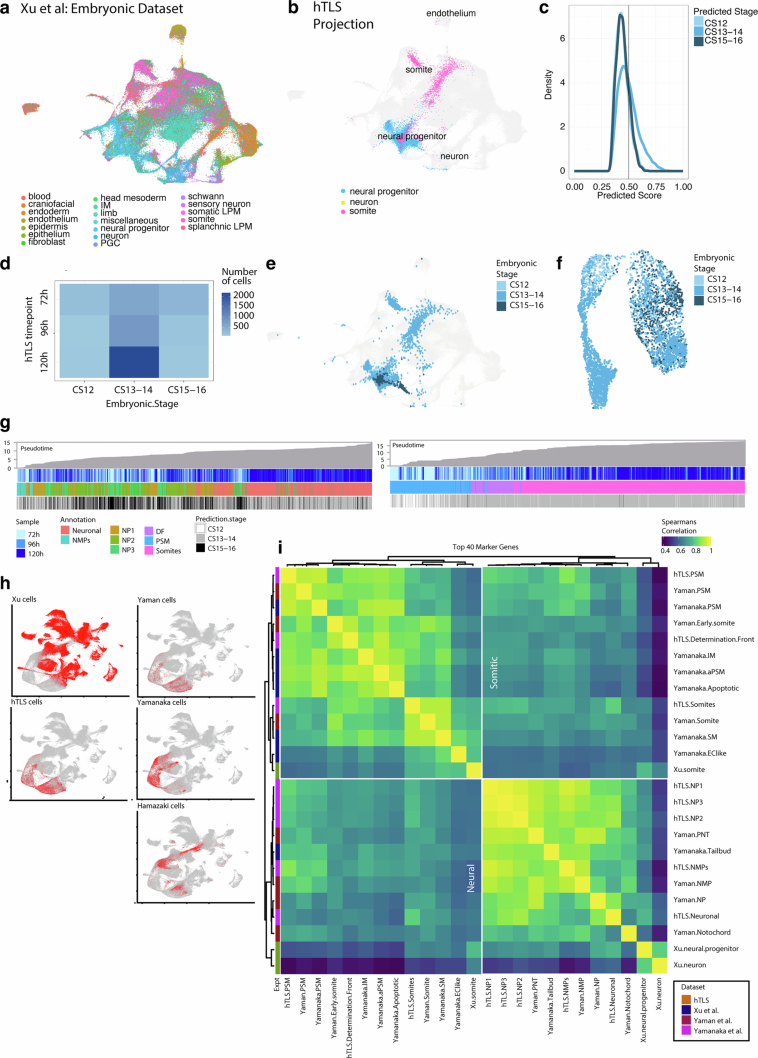
Bioinformatic comparison to a human embryo dataset. (**a**) UMAP projection of a human embryo atlas^2^. (**b**) Projection of hTLS single cell data onto embryo atlas, and coloured according to label transfer (pink, somite; blue, neural progenitor; yellow, neuron). (**c**) Density plot of the predicted confidence score for each projected cell. Vertical line shows the cutoff used for label transfer and subsequent analysis = 0.5. (**d**) Number of hTLS cells assigned to human embryonic stages by label transfer, across the analysed timepoints (72-120 h). (**e**) UMAP projection of hTLS cells onto human embryo atlas, coloured by equivalent embryonic stage according to label transfer. (**f**) UMAP projection of hTLS cells coloured by equivalent embryonic stage according to label transfer. (**g**) hTLS single cells ordered by pseudotime, showing sample, annotation and label transfer of equivalent embryonic stage. (**h**) Integration of multiple in vitro models^1,3,4^ with the embryonic dataset. Red indicates the position of cells from that dataset within the integrated UMAP (in grey). (**i**) Spearmans Correlation in gene expression between datasets from all time points. IM, Intermediate mesoderm; EC, Endothelial Cells; SM, Somitic mesoderm; PSM, Presomitic mesoderm; aPSM, anterior PSM; DF, Determination Front; NMPs, Neuromesodermal progenitors; NP, neural progenitor; MG, Matrigel; RAL, Retinal; PNT, Posterior Neural Tube; CS, Carnegie Stage. Source data

To further examine whether our annotated clusters had equivalents to the corresponding in vivo states, we projected our dataset onto a human embryonic atlas ranging from Carnegie stage (CS) 12 to CS16 (ref. ^38^). Our data clearly mapped to the somitic and neural progenitor populations, highlighting that the in vitro populations observed in the hTLS match the expected cell types in the human embryo (Fig. 1j and Extended Data Fig. 5a,b). Interestingly, when we performed label transfer for our dataset, the vast majority of cells were called as CS13–14 equivalent (day 28–35 of human development; Extended Data Fig. 5c–g), which is notably later than that predicted for human gastruloids (approximately CS8–9; day 17–21 of human development)^12^, indicating the substantial step forwards achieved by the hTLS protocol.

We also integrated the hTLS dataset with other in vitro protocols^15,21,22^ alongside the embryonic CS12–16 dataset^38^ for systematic comparison. We observed clear separation between in vitro and in vivo data in uniform manifold approximation and projection (UMAP) state space, particularly at early stages (Extended Data Fig. 5h), but overall unity of neural and somitic cell types across samples (Extended Data Fig. 5i), suggesting that, in general, these cell states approximated the embryonic identities of somitic and neural populations.

### Axial elongation in hTLS is maintained by an NMP population

**Extended Data Fig. 6:**
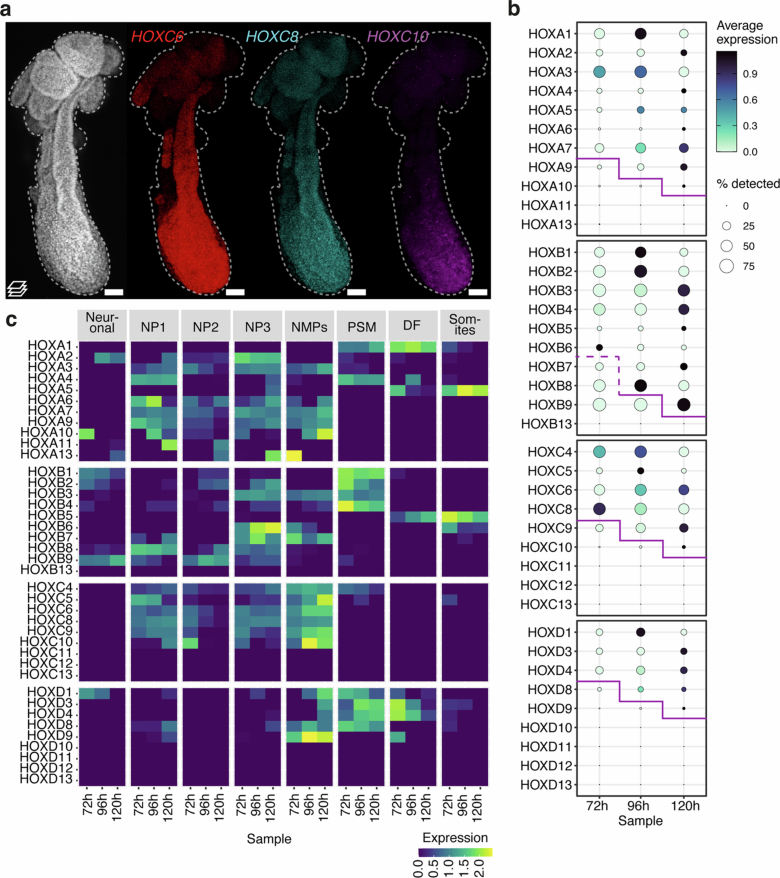
Spatiotemporal HOX gene expression. (**a**) Projected HCR images of whole mount 120 h hTLS. (*n* = 4) Scale bars, 50 μm. Dashed line outlines hTLS. (**b**) Temporal change in HOX gene expression from single cell transcriptomics. Purple line indicates the approximate maximal expression of HOX gene paralogues at each timepoint, dotted line indicates uncertainty in the HOXB cluster at 72 h timepoint due to low frequency of detection. (**c**) Spatiotemporal gene expression of HOX genes across single cell clusters. PSM, Presomitic mesoderm; DF, Determination Front; NMPs, Neuromesodermal progenitors; NP, neural progenitor. Source data

We next examined the spatial and temporal regulation of Hox gene expression, as these genes are known to be critical in the regulation of axial identity along the AP axis of vertebrate embryos^39^. We observed expression of *HOXC6/8* along the full AP axis of the hTLS, while *HOXC10* was only present in the posterior portion (Extended Data Fig. 6a). Temporally, there was progressive expression of HOX gene paralogues across the timepoints sampled (Extended Data Fig. 6b,c). Together with the lack of expression of HOX11–13, this suggests that the hTLS model approximates the thoracic and lumbar regions of the AP axis but not the sacral or caudal-most regions^40^. Given that termination of axial elongation is thought to be triggered by the expression of HOX13 in embryos^41,42^, we wondered what was mediating the extension and then termination of axial elongation in our hTLS model.

**Fig. 2:**
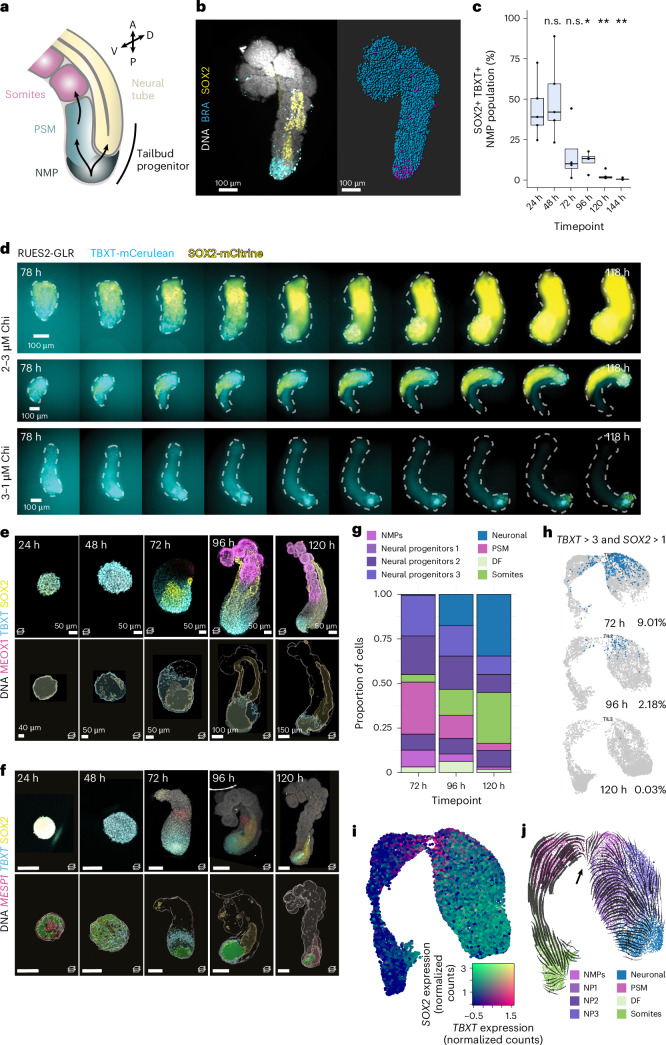
Dynamic NMP population in hTLS. **a**, Schematic of NMPs that contribute to both somites and neural tube tissues. **b**, Representative costaining of TBXT and SOX2 expression in 120 h H9-SBR hTLSs (left) and after nuclei segmentation (right) (blue, all nuclei; magenta, SOX2+ TBXT+ NMPs; *n* = 28; *Z*-step, 2 µm). **c**, Quantification of NMP numbers over time (*n* = 28). Box plots represent mean (centre line) and interquartile range (bounding box), with whiskers representing the largest and smallest value no further than 1.5× the interquartile range and outliers represented as black dots. n.s., non-significant. ^*^P < 0.05; ^**^P < 0.01 by two-sided Wilcoxon test (24–48 h: *W* = 11, *P* = 0.8413; 24–72 h: *W* = 17, *P* = 0.1111; 24–96 h: *W* = 20, *P* = 0.01587; 24–120 h: *W* = 25, *P* = 0.007937; 24–144 h: *W* = 25, *P* = 0.007937). **d**, Representative images of RUES2-GLR hTLSs imaged at 4h intervals over a period of 40 h. Cultures were pre-treated with 2 µM Chi and aggregated with 3 µM Chi (‘2–3’) or pre-treated with 3 µM Chi and aggregated with 1 µM Chi (‘3–1’). Dotted line outlines the brightfield images to show the full hTLS structure (*n* = 5–24). **e**,**f**, Staining of HES7::Achilles and LaminB1::mTagRFP-T hTLS (top) and Imaris-rendered regions of positive expression of TBXT (blue), SOX2 (yellow) and dual TBXT–SOX2 co-expression (green), by immunofluorescence (*n* = 5–11 per timepoint) (**e**) and HCR (*n* = 3–8 per timepoint) (**f**). *Z*-step, 2 µm. Scale bars, 100 µm. **g**, Proportion of cells within each cluster identity over time (*N* = 1). **h**, Percentage and location of double-positive (TBXT > 3 and SOX2 > 1 normalized counts) cells over time: 72 h (top), 96 h (middle) and 120 h (bottom). **i**, Co-expression of SOX2 and TBXT for hTLS cells projected onto a UMAP. **j**, RNA velocity map, showing vectorial directions of predicted differentiation. Black arrow indicates a branchpoint within the annotated NMP cluster. NMP, neuromesodermal progenitors; NP, neural progenitors; PSM, presomitic mesoderm; DF, determination front; D, dorsal; V, ventral. Source data

In the embryo, axis extension is supported by a population of NMPs: bipotential progenitors that give rise to both the neural and somitic cell fates^43–45^ (Fig. 2a). One basic characterization of NMPs is the co-expression of *TBXT* and *SOX2*^46^ that is thought to underlie their bipotency, and we observed cells co-expressing these markers in the posterior of the hTLS (Fig. 2b,c). Using the RUES2-GLR cell line^47^, which contains TBXT-mCerulean and SOX2-mCitrine fluorescent reporters, we live-imaged the hTLS under conditions that promoted co-development of the neural tube and somites (hTLS) or conditions that biased towards somite-only (high pre-treatment Chi conditions; for example, 3 μM Chi before treatment and 1 μM Chi at aggregation) or neural tube-only fates (low pre-treatment Chi conditions; for example, 2 μM Chi before treatment and 3 μM Chi at aggregation; Fig. 2d). We observed an early bias (between 78 and 90 h) towards SOX2-only or TBXT-only expression in neural tube-only and somite-only structures, respectively, with co-developing structures maintaining a TBXT and SOX2 co-expressing domain for the longest duration, consistent with the role of NMPs in contributing to both fates.

However, the NMP domain showed a clear decrease in volume over the hTLS time course, as was confirmed by staining at the messenger RNA and protein level (Fig. 2e,f) and by our single-cell RNA sequencing data (Fig. 2g–i). Indeed, RNA velocity analysis showed evidence of bi-directional fates predicted from this population (Fig. 2j), and trajectory analysis rooted in the NMP cluster predicted endpoints in both somites and neural tube populations (Supplementary Fig. 1). Together, these data suggest that hTLSs contain an NMP population capable of bipotential cell fate differentiation, and its gradual depletion may be responsible for the resultant maximum axial length and number of somites observed in hTLSs.

### Endogenous signalling patterns in hTLSs

**Fig. 3:**
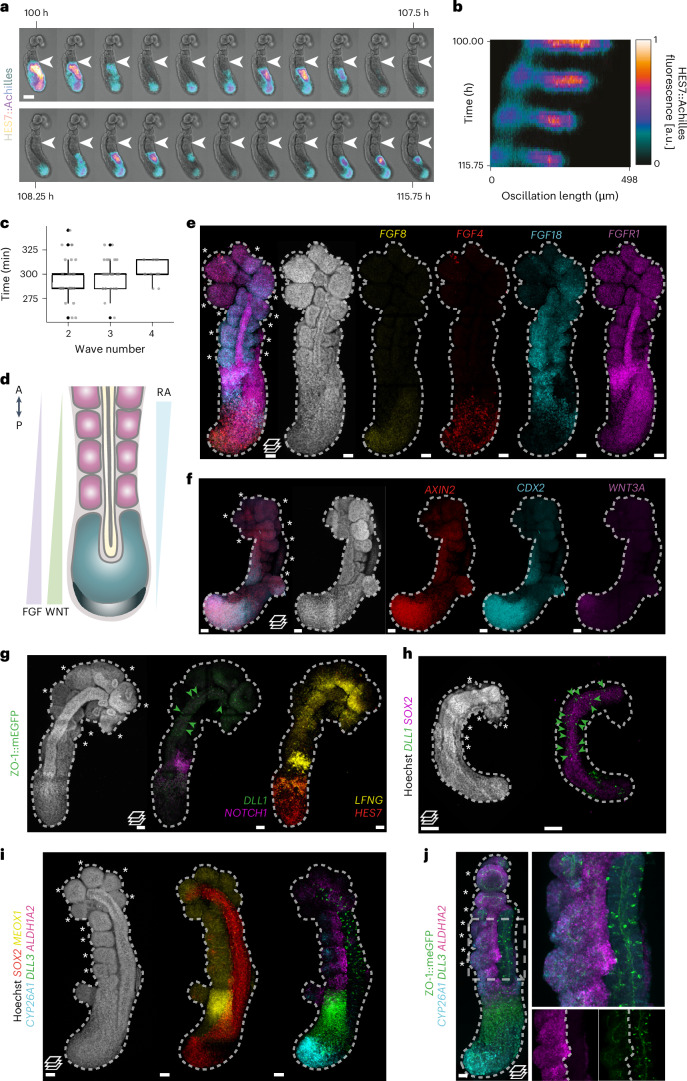
Characterizing signalling in hTLS models. **a**, Representative stills from live imaging of the HES7-Achilles hTLS at 15-min intervals. White arrows denote the same boundary, showing a posterior shift of the wavefront (*n* = 33). Scale bar, 100 µm. **b**, Kymograph of Achilles oscillations from the HES7::Achilles hTLS represented in **a**. **c**, Time intervals of HES7 oscillations from peak to peak (mean ± s.d.) (*n* = 33). **d**, Schematic of AP signalling gradients across the embryo axial length. **e**–**i**, Projected images of HCR staining of ZO-1::mEGFP hTLSs at 120 h for components of the FGF (*n* = 5) (**e**), WNT (*n* = 3) (**f**) and notch (*n* = 3) signalling pathways (**g**); expression of *DLL1* within the *SOX2*-expressing neural tube by confocal imaging (*n* = 3; scale bars, 100 μm) (**h**); and RA signalling pathway components along the AP axis (*n* = 2) (**i**). **j**, Localized mediolateral signal of *ALDH1A2* in somites and punctate signal of *DLL3* through the neural tube of ZO-1::mEGFP hTLS. The dashed box shows the location of the enlarged region (*n* = 4). Scale bars, 50 μm, unless otherwise stated. The green arrows show *DLL1* signal in the neural tube, the asterisks indicate somites and the dashed line outlines hTLS. A, anterior; P, posterior; RA; retinoic acid. Source data

In vertebrate embryos, the sequential addition of somites from the posterior unsegmented region is determined by a combination of local intrinsic oscillations of gene expression and a global signalling landscape^48^. Using a HES7::Achilles iPS cell line^49^, we observed oscillatory expression in the PSM of hTLSs, which moved posterior to anterior and stopped at the site of emergent somite formation (Fig. 3a,b and Supplementary Video 2), as has been described in embryos^50,51^. The time period of these oscillations comprised an average of 298 min (±17.2 s.d.; 4.96 h; Fig. 3c) which is consistent with in vitro measurements performed in alternative human systems (~5 h in somitoids^16^ and axioloids^15^ and 4.6 h in segmentoids^17^). However, an increased period of oscillations was observed for subsequent peaks, indicating that the clock is slowing down over time. This could be due to the exhaustion of the tailbud domain at these timepoints, similar to that seen in embryos at late somitogenesis^52,53^. Further research would be necessary to investigate the mechanism of this slowing.

We then turned our attention to the signalling landscape of the hTLS along the AP axis. In the embryo, the signalling gradients primarily responsible for the wavefront are thought to be fiboblast growth factor (FGF) and Wnt signalling in the posterior region and RA at the level of the somites (Fig. 3d). We observed *FGF8* and *FGF4* in the posterior region^54,55^; *FGF18* expression in the first three to five newly formed somites, the determination front and the anterior PSM^56^; and *FGFR1* in the neural tube and the determination front^54,57^ (Fig. 3e). We also saw posterior expression of *WNT3A* and *AXIN2* in the tailbud (Fig. 3f), consistent with a posteriorly localized Wnt signalling gradient^58–61^.

Likewise, we observed strong expression of Notch signalling ligands *DLL3* and *DLL1* in the anterior PSM and determination front and in a salt-and-pepper organization in the neural tube (Fig. 3g–i), matching descriptions in the embryo, where a Notch-dependent mutual inhibition mechanism leads to neural differentiation^62–64^. In addition, expression of *LFNG* and *NOTCH1* in the anterior PSM and newly formed somite is consistent with the role of Notch signalling in mediating the dynamic control of somitogenesis^65^ (Fig. 3g).

The crucial anterior signalling gradient that opposes the Wnt and FGF gradients is RA, which inhibits posterior FGF signalling and fine-tunes the progenitor-to-differentiated neuron gradient along the neural tube^8,66^. Typically, ALDH1A2 (an alcohol dehydrogenase involved in the synthesis of RA) and CYP26A1 (which degrades RA) have been used as a proxy for RA signalling status in chick^67^ and mouse embryos^68^. In the hTLS, *CYP26A1* is strongly expressed in the most posterior tip of the tailbud, with *ALDH1A2* expressed in the somites (Fig. 3i,j), which is indicative of a ‘source and sink’ pattern creating an AP gradient of RA signalling. As such, it is likely that self-organized signalling along the AP axis provides endogenous signals that establish the clock-and-wavefront mechanism of somitogenesis in hTLSs.

### Exogenous SHH defines the DV identity of hTLS structures

In the embryo, signals from the surrounding tissues, including the notochord, lateral plate mesoderm and surface ectoderm, are all integrated to provide positional information to cells within the neural tube and somites^69^. In embryo models, the presence or absence of such tissues can allow us to investigate the extent of positional information in a simplified system. For instance, SHH signals from the notochord play a key role in setting up the dorsoventral (DV) axis in both the neural tube and somites (see ref. ^70^ for review). We therefore wondered what DV identity our hTLS structures assumed in the absence of these tissues.

**Fig. 4:**
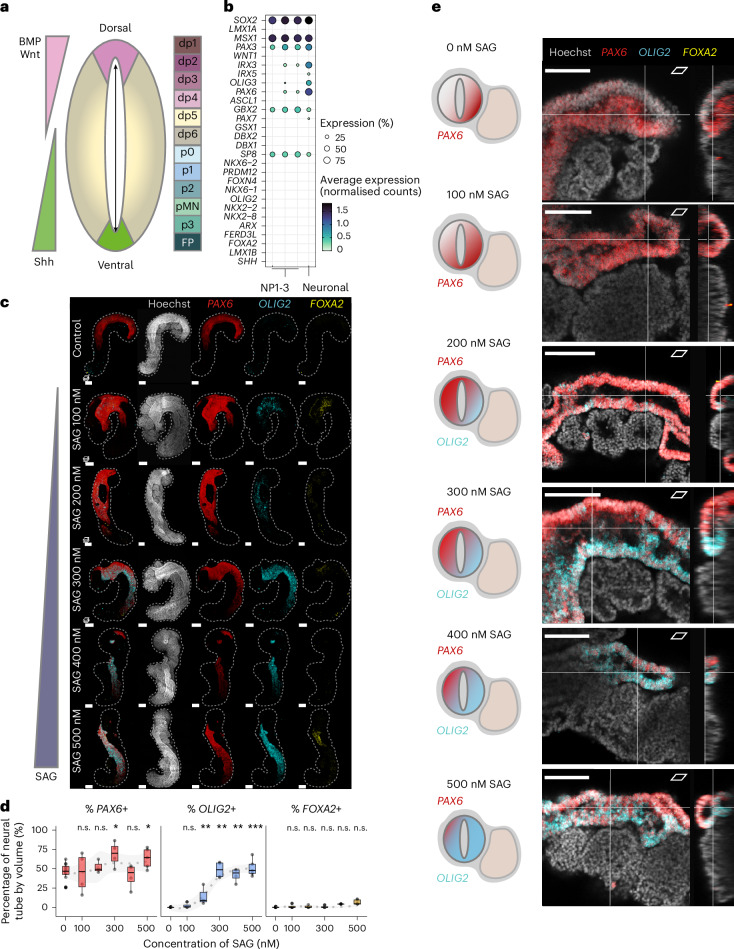
DV patterning in hTLS structures. **a**, Schematic diagram of the signalling gradients (left) and subsequent DV patterning of the neural tube (middle), leading to discrete neural populations (right). BMP, bone morphogenetic protein. **b**, Gene expression of neural populations in hTLS, ordered from top to bottom by expression along the DV axis in embryos. **c**, Projected images of 144-h LaminB1::mTagRFP-T hTLS treated with increasing concentrations of SAG (SHH pathway activator) and HCR stained for neural markers (*n* = 5–8 per condition). Dashed line outlines hTLS boundaries. Scale bars, 100 µm. **d**, Quantification of expression by volume (%) with increased exposure to SAG. Dashed line indicates smoothed trend with 0.95 confidence interval (grey ribbon). Box plots represent mean (centre line) and interquartile range (bounding box), with whiskers representing the largest and smallest value no further than 1.5× the interquartile range and outliers represented as black dots. n.s., non-significant. ^*^P < 0.05; ^**^P < 0.01 obtained by two-sided Wilcoxon test compared with 0 µm SAG control (*n* = 3–12 per condition). **e**, Schematic diagrams (left), *XY* images (middle) and *YX* optical slices (right) showing HCR gene expression in the 144-h hTLS neural tube with increasing SAG concentrations. Scale bars, 150 µm for SAG 200 nM condition and 100 µm for all others in **e**. Source data

**Extended Data Fig. 7:**
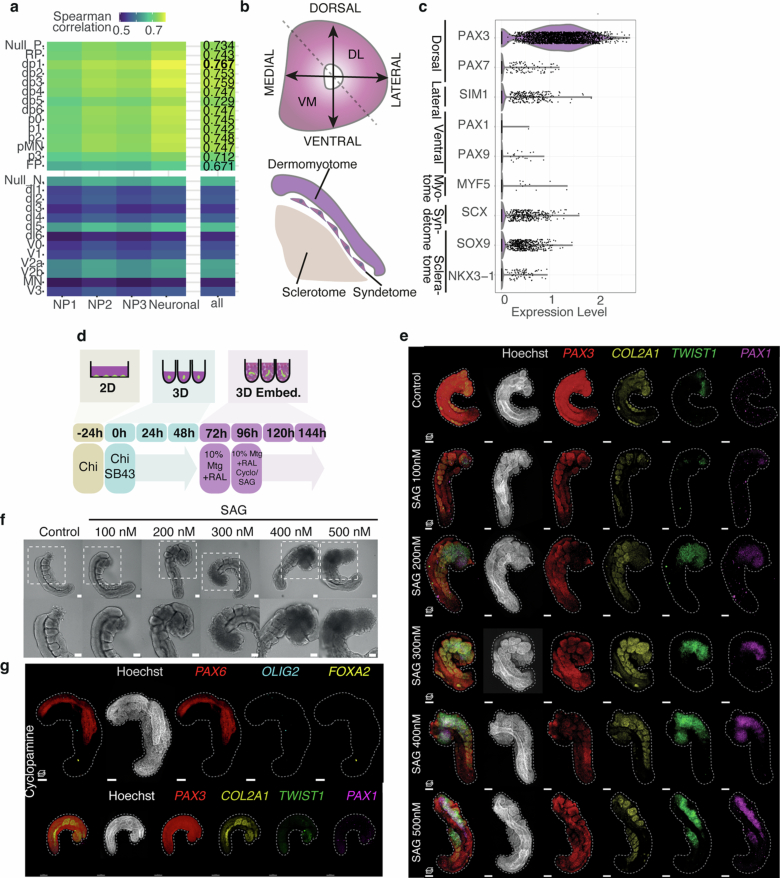
Dorsoventral patterning in hTLS. (**a**) Correlation coefficients between neural populations in 120 h hTLS and the human embryonic neural tube^5^. NP, Neural progenitor; Null_P, unannotated progenitor; RP, Roof plate; dp, Dorsal progenitor; p, Progenitor; pMN, Motor Neuron progenitor; FP, Floor plate. (**b**) Schematic diagram of the DV and mediolateral axes of the developing somite (top) and subsequent tissue maturation (bottom). (**c**) Gene expression signature of somites in hTLS. (**d**) Schematic diagram of hTLS treatment with SAG (sonic hedgehog (SHH) pathway activator) and cyclopamine (SHH pathway inhibitor). Chi, Chrion (CHIR99021); Mat, Matrigel; SB43, SB431542; RAL, retinal. (**e**) Projected images of HCR staining in 144 h hTLS with increasing SAG exposure (*n* = 5-8 per condition). (**f**) Representative brightfield images of SAG-treated hTLSs at 144 h. Dashed box represents position of enlarged region. (*n* = 58-70 per condition) Scale bars, 100 μm. (**g**) Projected images of HCR staining in 144 h hTLSs treated with cyclopamine (*n* = 6-8). Dashed outline hTLSs. Scale bars, 100 µm. Source data

On the basis of transcriptomic data analysis, we found that the hTLS neural tube has a dorsal identity (*PAX6*, *OLIG3* and *IRX3)*, with no detectable ventral (*OLIG2* and *NKX2-2/8*), floor plate (*FOXA2* and *SHH*) or roof plate markers (Fig. 4a,b). Indeed, comparison of the hTLS neural clusters with human embryonic spinal cord data^71^ showed that they aligned the most strongly to progenitor dorsal cell identities, excluding the roof plate (Extended Data Fig. 7a).

Likewise, the somitic identity (Extended Data Fig. 7b) of hTLS show expression of pan-somite markers (*TCF15*, *MEOX1* and *FOXC2*), dorsal markers of dermomyotome (*PAX3*, *PAX7*, *SIX1*, *SIX4* and *EYA1*) and syndetome (*SCX*; Extended Data Fig. 3c,h), with very few or no late dermatome or myotome markers (*MYF5*; Extended Data Fig. 7c; *MYOD* and *MYOG* not expressed). We observed few cells expressing markers of ventral somitic identity, the sclerotome, with only a handful expressing detectable *PAX1* or *PAX9* (Extended Data Fig. 7c). Together, this suggests that the lack of notochord leads to an overall dorsalization of both the somites and the neural tube in hTLSs.

To test whether hTLSs do still have the capacity to ventralize, we exposed the structures to varying concentrations of Smoothened agonist (SAG), which promotes SHH signalling, as well as to the small molecule cyclopamine that inhibits SHH signalling^72^ as a control (Extended Data Fig. 7d). Upon SAG addition, we observed increasing ventralization of hTLS structures in a dose-dependent manner, with increased expression of ventral neural tube (*OLIG2* and *FOXA2*; Fig. 4c) and ventral somite (*PAX1* and *TWIST1*) markers (Extended Data Fig. 7e). In addition, the morphology of hTLSs appeared to be altered under increasing SAG exposure with less compacted anterior somites (Extended Data Fig. 7f), suggesting that epithelial-to-mesenchymal transition associated with ventral (sclerotome) fates could be occurring, while cyclopamine had no observable effect on hTLSs (Extended Data Fig. 7g). However, the dose-dependent ventralization of hTLSs exposed to SHH signalling was not simply all-or-nothing. Surprisingly, we observed that at high concentrations of SAG exposure, not only did proportions of *OLIG2*+ cells increase in the neural tube (Fig. 4d), but these were organized across an axis orthogonal to the AP axis (Fig. 4e). In the embryo, it is known that negative feedback between transcription factors acts to establish discrete domains of neural progenitor identity along the continuous SHH gradient^73^, and the same is probably happening in the hTLS model. However, because the hTLSs are exposed to a single, exogenous concentration of SAG throughout (rather than a localized source), the appearance of a consistent axis was surprising. In this case, we reasoned that the juxtaposition of the somites next to the neural tube may establish some aspects of an axis because the *OLIG2*+ domain was localized adjacent to the somites (Fig. 4c–e). We therefore set out to investigate the nature of this communication between the somites and the neural tube.

### Signalling interactions between tissues

**Fig. 5:**
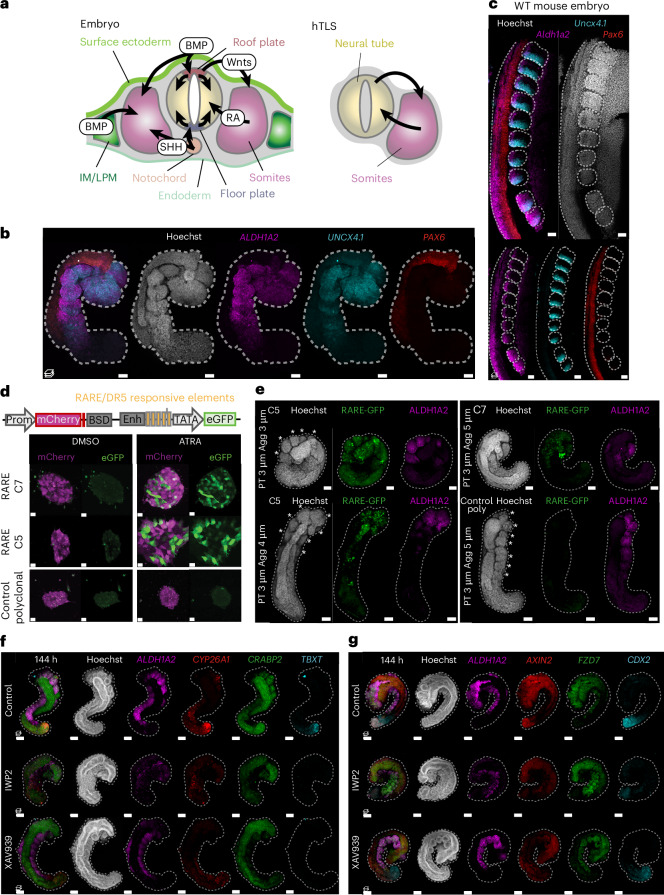
Interactions between somites and the neural tube in hTLS and embryos. **a**, Schematic diagram of the reciprocal signalling environment of the embryo (left) and hTLS (right). RA, retinoic acid; IM, intermediate mesoderm; LPM, lateral plate mesoderm. **b**,**c**, Somite-specific *ALDH1A2* expression adjacent to the neural tube, in 120 h ZO-1::mEGFP hTLS (*n* = 4) (**b**) and E9.5 mouse embryo (*n* = 3) (**c**). **d**, Schematic of the RARE reporter cassette (top; diagram adapted from the LipExoGen website) and its functional validation upon RA addition in 2D cultures of clonal (C5 and C7) and polyclonal DR5/RARE reporter (middle) and control cells lacking the DR5/RARE elements (bottom). Prom, promoter; BSD, blasticidin selection domain; Enh, enhancer. **e**, The 120-h hTLS structures made with RARE reporter cell lines or control lines, with the addition of ALDH1A2 antibody staining (*n* = 1–8, *Z*-step, 2 µm and averaging eight images). **f**,**g**, Wnt signalling inhibition through IWP2 and XAV393 and resulting effect on elements of the RA (**f**) or Wnt (**g**) signalling pathways in 144-h LaminB1::mTagRFP-T (*n* = 6–8 per condition). Scale bars, 50 μm (**b**–**e**), 100 μm (**f** and **g**). Asterisks indicate somites; the dashed line outlines the hTLS.

One of the primary benefits of a co-developing system such as the hTLS is the ability to use it to probe the interactions between tissues, which would otherwise present challenges in an embryonic context. Beyond the difficulties of accessing human embryonic material at these stages of development, the complexity of the signalling landscape—with signals emanating from diverse tissues and organs, across multiple spatial axes and dynamically changing over time—makes it complicated to unravel the contribution of a single tissue. In this case, we were interested in the signalling interactions between the neural tube and somites, in the absence of all other tissues (Fig. 5a).

**Extended Data Fig. 8:**
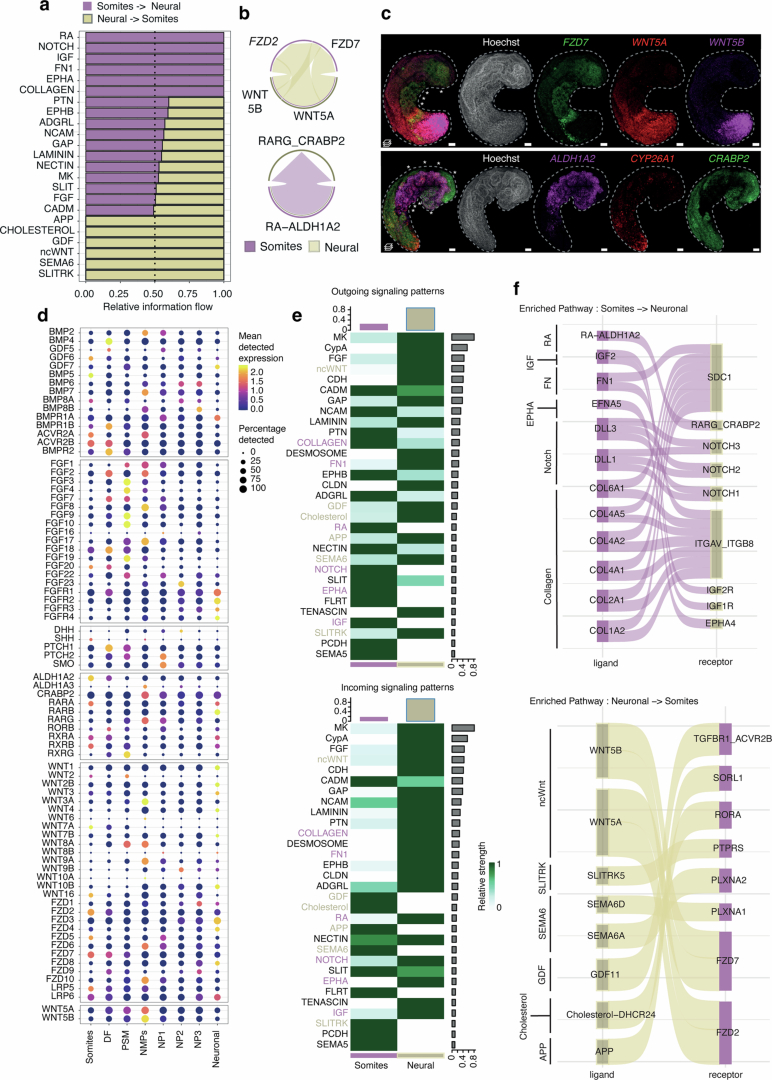
CellChat Analysis. (**a**) Relative information flow from CellChat^6^ analysis, identifying unique signalling interactions between the somite cell cluster and neural clusters (combined Neural progenitor populations 1-3 and Neuronal clusters). Dotted line indicates equal information flow = 0.5. (**b**) Predicted signalling direction of non-canoncial Wnt and RA pathways, as predicted by CellChat. (**c**) Localised expression of non-canonical Wnt (top; *n* = 3) and RA (bottom; *n* = 2) signalling components by HCR in 120 h LaminB1::mTagRFP-T hTLS. Scale bars, 50 μm. (**d**) Gene expression of signalling ligands and receptors at single cell level across populations. (**e**) CellChat analysis showing statistically significant interactions between the somite and neuronal populations. Pathway names coloured in purple indicate possible somite-to-neural directional signalling, and names coloured in ochre indicate possible neural-to-somite directional signalling. (**f**) CellChat-predicted interactions between ligands and receptors, organised by pathway. Source data

We turned first to unbiased, computational approaches to interrogate the putative signalling ligand–receptor interactions predicted between the two tissues. Building on the expression profile of ligands and receptors of known pathways, we used CellChat^74^ to identify statistically significant, unidirectional interactions specifically from the somitic to the neural tissues (combining neural progenitor populations 1–3 and neuronal clusters) or the inverse interaction. This identified several putative signalling interactions, including RA, Notch, IGF, fibronectin and EphA signalling from somites to neural tissue and cholesterol, GDF, non-canonical Wnt, SEMA6 and SLITRK pathway signalling from neural to somitic tissues (Extended Data Fig. 8).

We validated the expression signatures of somite-derived RA and neural tube-derived non-canonical Wnt signalling by examining the localization of the gene corresponding to an RA-binding protein, *CRABP2* (alongside *ALDH1A2* and *CYP26A1*), which was expressed in the neural tube, as predicted, and by expression of ligands *WNT5A* and *WNT5B* in the neural tube and receptor *FZD7* in the somites (Extended Data Fig. 8c). We therefore hypothesized that signalling between the two tissues in the hTLS is responsible for setting positional identity.

### Mediolateral ALDH1A2 expression in somites

**Extended Data Fig. 9:**
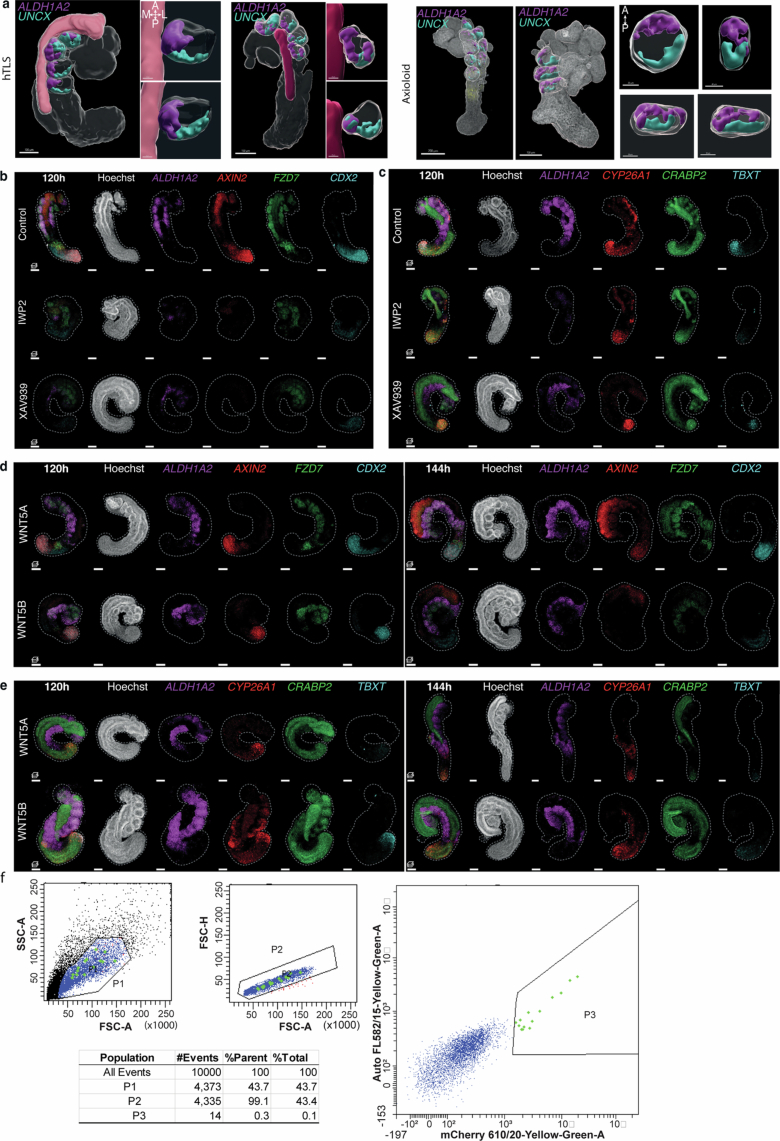
Medially localised ALDH1A2 expression in somites is established by non-canonical Wnt signalling. (**a**) Imaris-rendered surfaces showing regions of *ALDH1A2* (purple) and *UNCX* (blue) expression by HCR within somites, relative to the position of the neural tube (pink), for both hTLS (left) and axioloid (right) structures made from ZO-1::mEGFP cells. A, anterior; P, posterior; M, medial; L, lateral. (**b**–**e**) Projected HCR images of whole mount 120 h and 144 h hTLS structures exposed to Wnt signalling inhibition. Scale bars, 100 μm (*n* = 5-8 per condition). (**f**) FACS gating strategy for single-cell sorting for the derivation of monoclonal transgenic cell lines, shown for FCI-GM25256. First, cells were gated based on size and granularity (SSC-A vs. FSC-A). From this population, singlets were identified using FSC-H vs. FSC-A. Finally, Cherry-positive cells were selected for sorting (gate P3). One Cherry-positive cell per well was sorted to establish monoclonal cell lines.

An additional surprise came on closer examination of the somites, where we noticed that *ALDH1A2* was not uniformly localized across the somite but instead was most strongly expressed adjacent to the neural tube (Fig. 5b and Extended Data Fig. 9a). This is in direct contrast with somite-only axioloid structures, where ALDH1A2 was shown to instead have an AP polarity^15^, consistent with our own experiments in axioloids (Extended Data Fig. 9). This hinted that the presence of the neural tube may bias the localization of *ALDH1A2* expression to the neural tube-adjacent side, with important implications for the mechanism of mediolateral polarization in somites. Although this phenomenon does seem to be apparent from closer inspection of existing data^75^, we examined the same gene expression in E9.5 mouse embryos (Fig. 5c), where we likewise observed a clear medial bias of *ALDH1A2* expression in the somites (Supplementary Video 3).

To verify that endogenous RA signalling was indeed occurring in hTLSs, we engineered a RARE/DR5–eGFP reporter line (where DR5 is direct-repeat-5) that contains five RARE/DR5 responsive element repeats that promote eGFP expression as a result of RA binding (FCI-GM25256-RARE), or control reporters without the RARE binding sequence (FCI-GM25256-Ctrl; Fig. 5d and Extended Data Fig. 9f). Although spontaneous reporter silencing led to a salt-and-pepper pattern of fluorescence, clear signal could be seen in both the neural tube and somites, indicating that active RA signalling is indeed occurring in the system (Fig. 5e). We also made use of an ALDH1A2 antibody to confirm protein-level expression of the enzyme in the somites of hTLS structures, although the medial bias was less clear at the protein level (Fig. 5e).

Together, these data suggest that the neural tube biases the localization of ALDH1A2 to the medial somite domain in both embryos and models, which leads to active RA signalling exposure within both the somites and the neural tube.

### Non-canonical Wnt signalling establishes *ALDH1A2* expression in somites

It remained unclear how the medially localized *ALDH1A2* expression was established in somites. We reasoned that it was probably due to signalling from the neural tube, given the localization adjacent to the tube, and so turned to the other significantly enriched pathway proposed via CellChat analysis (Extended Data Fig. 8), the non-canonical Wnt (ncWnt) signalling pathway through WNT5A and WNT5B. To investigate whether ncWnt might act upstream of *ALDH1A2* localization, we inhibited signalling using either IWP2 (a porcupine inhibitor targeting both canonical and ncWnt) or XAV939 (a tankyrase inhibitor targeting only canonical Wnt signalling) or activated it with recombinant WNT5A or WNT5B. While XAV393 had a mild effect on hTLS structure at 120 h (Extended Data Fig. 9b,c), this had fully resolved by 144 h, which was equivalent to the control structures (Fig. 5f,g). However, IWP2 had a sustained impact on both *ALDH1A2* expression and hTLS morphology, with a lack of somitic epithelialization that mirrored the effect of the lack of retinal (Fig. 5f,g and Extended Data Fig. 1i). WNT5A and WNT5B had little discernible effect on either the morphology or patterning of hTLS structures (Extended Data Fig. 9d,e). While we cannot rule out that the lack of epithelialization could be a cause rather than a consequence of the lack of *ALDH1A2* expression, it is possible that ncWnt is the signal originating in the neural tube that leads to localized ALDH1A2, which in turn drives endogenous RA signalling and subsequent epithelialization.

### Somite-to-neural tube RA signalling in hTLS

We next turned our attention to the downstream role of localized *ALDH1A2*. In the embryo, RA signalling from the somites to the neural tube is thought to have three main functions: (1) AP axis organization and morphogenesis of the neural tube, (2) promoting cellular neurogenesis and (3) mediating the DV patterning of neural tube populations (see ref. ^76^ for review). In addition, in the absence of RA signalling, there is an expanded domain of ventral gene expression in the neural tube^77^, suggesting that it has a dorsalizing influence on the neural tube.

**Extended Data Fig. 10:**
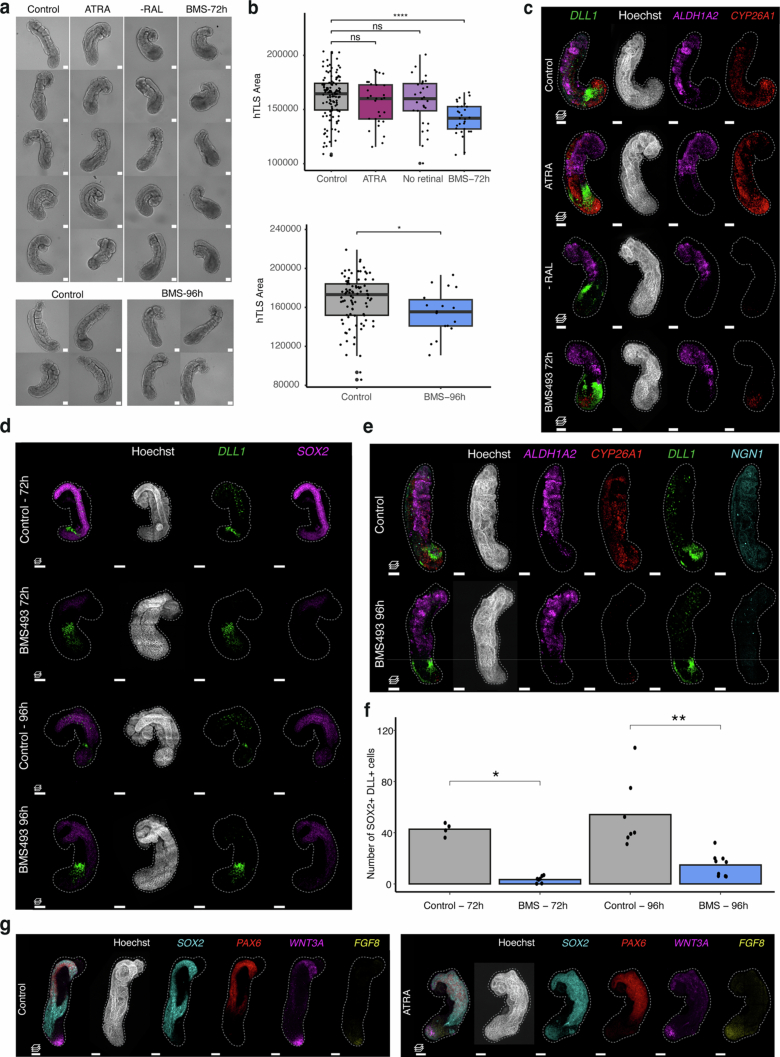
Effect of retinoic acid exposure and inhibition on hTLS. (**a**) Representative brightfield images of 120 h LaminB1::mTagRFP-T hTLS with replacement of RAL with all *trans*-retinoic acid (ATRA), without retinal (RAL), or with treatment with BMS493 (RA inhibitor) at 72 h (top) and 96 h (bottom). Scale bars, 50 µm. (*n* = 29-135 per condition) (**b**) Quantification of hTLS area following RA signalling modulation. Box plots outline the mean (horizontal line), lower and upper quartiles (box hinges), 1.5x interquartile range (whiskers) and outlying datapoints (full circles). * indicates *P* < 0.05 and **** indicates *P* < 0.0001 obtained by two-sided Wilcoxon test between control and ATRA (W = 1536, p = 0.1647), No Retinal (W = 2033, p = 0.3842) and BMS-72h (W = 722, p = 3.439e^−7^), or control and BMS-96h (W = 552, p = 0.03378) (*n* = 18-127 per condition). (**c**) Projected images of 120 h LaminB1::mTagRFP-T hTLS HCR-stained following RA signalling pathway modulation. (*n* = 4-8 per condition). Dashed lines outline hTLS. (**d**, **e**) Effect of RA modulation on neurogenesis as assessed by *DLL1* + HCR staining in the neural tube of 120 h LaminB1::mTagRFP-T hTLS. (*n* = 2-7 per condition) (**f**) Quantification of *SOX2* + *DLL1+* cells by HCR. * indicates *P* < 0.05 and ** indicates *P* < 0.005 obtained by two-sided Wilcoxon test between the control and treatment groups at 72 h (W = 28, p-value = 0.01038) and 96 h (W = 62, p-value = 0.001436; n = 27). (**g**) Effect of ATRA addition on 120 h LaminB1::mTagRFP-T hTLS, including *PAX6* expression. (*n* = 3-5 per condition). RA, Retinoic acid; ATRA, all-trans Retinoic Acid; RAL, Retinal. Scale bars, 100 µm, unless otherwise stated. Source data

To test whether similar effects were also occurring in hTLS structures, we prevented RA signalling either by removal of the exogenous retinal or by exposure to the pan-RA receptor (RAR) inhibitor, BMS493^78^. Following early (72–120 h) exposure to BMS493, hTLS structures were morphologically impaired, with a smaller overall area, and were composed of fewer organized somites, a smaller neural tube and an increased tailbud size (Extended Data Fig. 10a,b). This is consistent with the role of RA signalling in axial organization and morphology, as *Raldh2*^−/−^ mouse embryos die mid-gestation with aberrant morphology that includes a truncated posterior axis, small somites and an irregularly folded neural tube^79^. However, when hTLSs were exposed to BMS493 at later timepoints (96–120 h), there was a significant reduction in area but overall morphology was comparable to controls (Extended Data Fig. 10a,b). This suggests that at this point, morphology had already been established and blocking the RA signalling might specifically affect the ongoing interactions between somites and the neural tube. Indeed, hTLS structures exposed to BMS493 at either timepoint showed much lower or no *CYP26A1* in the neural tube, in contrast to control structures (Extended Data Fig. 10c) consistent with published data from chick embryos^77^. We therefore further investigated the proposed role of somite-derived RA signalling on the neural tube by focussing on the latter timepoint.

To investigate the role of somite-derived RA on early neurogenesis, we examined marker expression in hTLS structures, including *DLL1* and *NGN1* (Fig. 3g and Extended Data Fig. 10d–f). Upon RA signalling inhibition, we observed significantly fewer *DLL1*+ cells in the neural tube (Fig. 5f). In vitamin A deficient (VAD) quails that lack the precursor to RA, retinal, *Delta-1* expression is depleted from the caudal neural tube in 9–19 somite-stage embryos^8^, but in the *Raldh2*^−/−^ mouse model, *Delta-1* expression is comparable to wild type (WT) at the 14–15 somite stage^80^. As such, the observation that hTLSs show RA-dependent neurogenesis hints that there may be additional species-specific differences in how RA signalling affects the neurogenesis cascade.

**Fig. 6:**
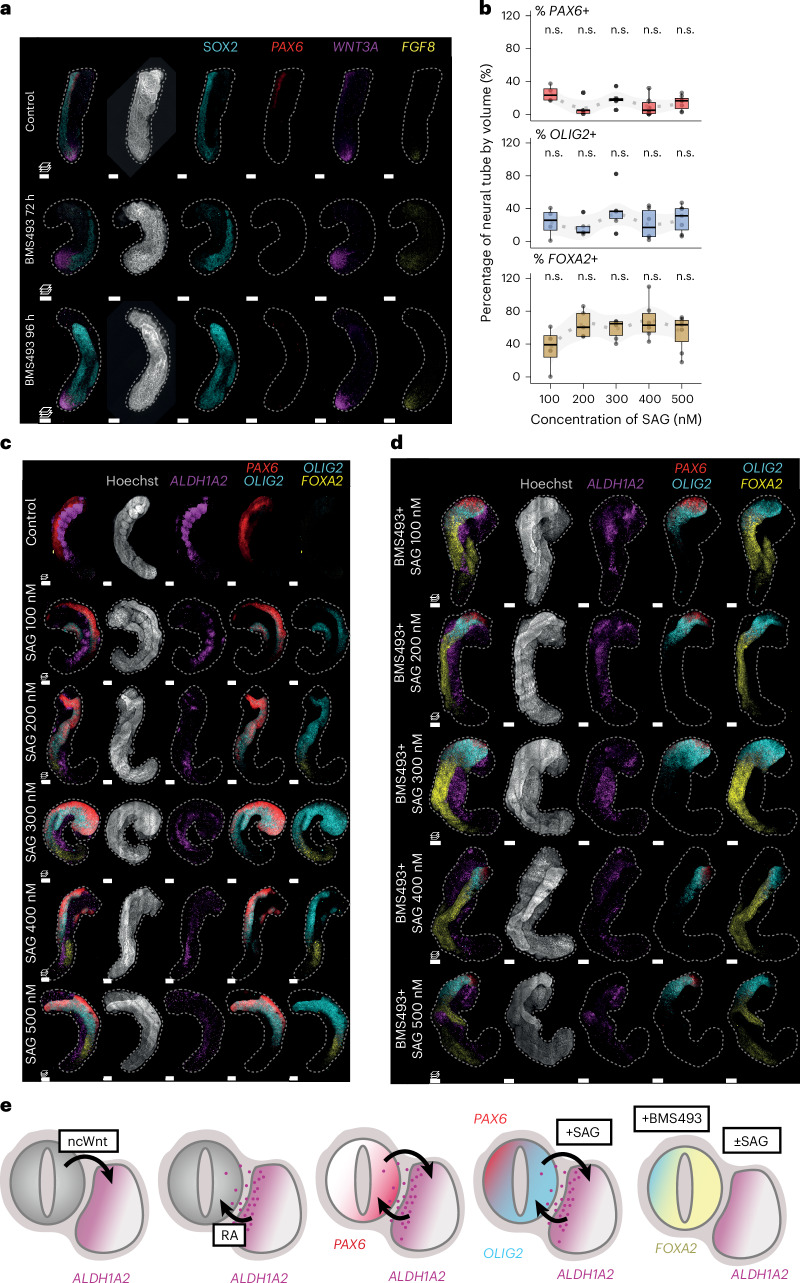
Effect of combined RA and SAG modulation on hTLS. **a**, LaminB1::mTagRFP-T 120 h hTLS structures following RA inhibition with BMS493, showing depletion of *PAX6* expression from the neural tube (*n* = 3–5 per condition). **b**, Quantification of HCR expression by neural tube volume (%) with exposure to SAG and BMS493. The dashed line indicates the smoothed trend with 0.95 confidence interval (grey ribbon). The box plots represent mean (centre line) and interquartile range (bounding box), with whiskers representing the largest and smallest value no further than 1.5× the interquartile range and outliers represented as black dots. n.s., non-significant. ^*^P < 0.05; ^**^P < 0.01 obtained by two-sided Wilcoxon test compared with all samples. **c**,**d**, Effect of variable SAG exposure without (**c**) (*n* = 5–6 per condition) and with (**d**) RA inhibition by BMS493 on 144 h LaminB1::mTagRFP-T hTLS (*n* = 5–7). **e**, Schematic diagram of proposed reciprocal signalling occurring between somites and the neural tube of hTLSs. Non-canonical Wnt signalling from the neural tube probably biases *ALDH1A2* expression in the somites towards the neural tube (far left), followed by subsequent RA signalling from the somites to the neural tube (middle left). This leads to *PAX6* expression being biased towards the somitic side (middle), or in the context of SAG exposure, increased *OLIG2* expression adjacent to the somites (middle right). This patterning is dependent on endogenous RA signalling because inhibition leads to *FOXA2* expression in the absence of other mediolateral patterning (far right). Asterisks indicate somites; dashed lines outline the hTLS. Scale bars, 100 μm. RA, retinoic acid; IM, intermediate mesoderm; LPM, lateral plate mesoderm. Source data

We next examined the role of somite-derived RA signalling on patterning in the neural tube. It is known that *Raldh2*^−/−^ mice and VAD quails have depleted expression of *Pax6* in the neural tube compared with WT embryos^75,77^. We therefore examined the expression of *PAX6* in the hTLS with late RA signalling inhibition and observed a substantial depletion of this neural marker from the neural tube (Fig. 6a). Conversely, the addition of all-*trans* RA (ATRA) onto hTLS structures increased the expression of *CYP26A1* in the neural tube, with no obvious difference in *DLL1* expression and with broad expression of *PAX6* across the neural tube (Extended Data Fig. 10g). Taken together, this suggests that somite-derived RA signalling is required for *PAX6* expression in hTLS and, given the spontaneous neural tube patterning observed (Fig. 4), makes it likely that RA signalling is responsible for establishing this patterning. Similar SHH-independent RA signalling effects on neural progenitor identity have been shown in chick embryos^81^ but not in a human system.

To test this hypothesis, we performed an experiment in which we varied the concentration of SAG exposure in the presence of BMS493 to inhibit RA signalling. The logic was that if endogenous RA signalling from somites was responsible for the localized induction of *OLIG2* with dose-dependent exposure to SAG, these expression patterns should be abrogated when an RA signalling inhibitor was added. Indeed, we observed decreased *PAX6* expression and a marked increase in *FOXA2* expression under BMS493 treatment (compare Figs. 4d and 6b) across all SAG exposures, while OLIG2 was no longer dose-responsive to SAG (Fig. 6b–d). As transcription factor networks composed of negative feedback motifs have been well described in the patterning of the neural tube^82,83^, it is likely that the concomitant increase in *FOXA2* and decrease in *PAX6* is the direct result of feedback mechanisms due to the lack of RA-responsive gene expression. The lack of dose-responsiveness to SAG also indicates that RA is necessary for fine-tuning the neural identities, while the consistent presence of PAX6+ and OLIG2+ cells always at the anterior part of the neural tube (Fig. 6d) indicates that timing of exposure to, or inhibition of, RA/SHH signalling likewise plays a role in patterning. This result showcases the flexibility of using an in vitro model to explore the interplay between endogenous signalling, intrinsic regulatory networks and exposure to external signalling factors, to better understand patterning dynamics, especially in a human context.

Taken together, these data suggest that initial ncWnt signalling from the neural tube to the somites results in the medial somitic localization of ALDH1A2 adjacent to the neural tube, which, in turn, leads to RA signalling from somites to the neural tube that promotes *PAX6*/*OLIG2* expression specifically at the side adjacent to the somites (Fig. 6e). Integration with the SHH pathway leads to progressive ventralization while maintaining the self-organized patterning in the neural tube established by localized signalling from the somites, due at least in part to endogenous RA signalling. This reciprocal signalling between the somites and the neural tube may therefore play an important role in ensuring that correct axial patterning is coordinated between tissues and suggests that elements of a mediolateral or DV axis can be established, even in the absence of signalling centres such as the notochord or lateral plate mesoderm.

## Discussion

Our system has revealed several features associated with the development of the paraxial mesoderm and neural tube. Interestingly, although hTLSs represent the thoracic and lumbar region, the neural tube structures develop through a process similar to secondary neurulation, which is characteristic of the tail region^32^. This suggests that, in the embryo, the mode of neurulation is dependent on the early morphology at gastrulation, and because the hTLS model bypasses these morphological stages, they utilize secondary neurulation mechanisms instead. This may also explain why different species evolutionarily rely on primary and secondary neurulation to different extents^84^, as a function of their early morphological constraints. It would be interesting to explore in subsequent research whether this substitution in the mode of neurulation plays a role on the cell biology or fate decisions in the neural tube.

The most noteworthy findings from this work concerns signalling interactions between the paraxial mesoderm and the neural tube. For instance, the hTLS model revealed medially localized *ALDH1A2* in the early somites of both hTLS and E9.5 mouse embryos. It could be that this organized gene expression is necessary for subsequent differentiation into the epaxial and hypaxial muscles^85^ and may represent the earliest distinction of mediolateral identity in the somites. It also demonstrates that neural tube-derived signals are sufficient to initiate mediolateral patterning in the somites, even in the absence of the intermediate or lateral plate mesoderm that would normally be localized laterally.

We also identified spontaneous patterning of the neural tube orthogonal to the AP axis, on the basis of somite-adjacent localization and due to RA-induced signalling emanating from the somites. While this is likely to reflect RA dependency rather than DV axis patterning per se, somite-derived signals in the human embryo could certainly contribute to DV axis patterning alongside the influence of the notochord. Previous research in animal models has suggested that RA signalling indeed plays an important role in DV patterning, but its precise function is often difficult to isolate owing to the complex interactions with other signalling pathways, including the SHH, Wnt and FGF pathways. For instance, VAD quail embryos exhibit a ‘dorsal shift’ in neural tube patterning, with expanded domains of genes associated with a ventral identity compared with WT^77^, suggesting a dorsalizing role for RA signalling. This is complementary to the results we describe following RA signalling inhibition under SAG exposure, where hTLS neural tubes are ventralized. Embryo models such as hTLSs may therefore allow us to isolate pathways, disentangling the roles of different signalling mechanisms in tissue patterning.

However, while the hTLS model offers many advantages, it is important to recognize its limitations. For example, our data suggest that the notochord is not strictly required for neural tube-derived signalling to the somites, but we cannot exclude the possibility that additional relay interactions, such as notochord-induced floor plate maturation, may additionally occur in the embryo. We also cannot rule out that some of the differences between somite-only (axioloids) and somite and neural tube-containing hTLS might be partially due to differences in Chi concentration exposure. Dissection experiments where tissues are removed after specification may resolve this by decoupling the signalling induction from tissue presence or absence but would be technically challenging. These considerations highlight the distinction between embryo models and embryos, emphasizing the complementary role of such models in developmental research.

Moreover, we anticipate that hTLS models could be of value in disease modelling in the future, given their origin from human iPS cells. One notable observation from our study is the formation of the hTLS neural tube via a secondary neurulation-like process, which could prove valuable for investigating rare NTDs.

Overall, we have shown that interactions between the somites and the neural tube leads to patterning events including mediolateral *ALDH1A2* expression in somites and patterning in the neural tube, mediated through localized RA signalling. These events are spontaneous and self organized, and they can enable us to explore how feedback and relay mechanisms combine to enable co-development across tissues in a human developmental context.

## Methods

### Ethical statement

The hTLS model as described in this manuscript represents a human stem cell-based embryo model of post-gastrulation stages. However, the model lacks extraembryonic tissues (and is therefore ‘non-integrated’ by 2021 International Society for Stem Cell Research (ISSCR) terminology on embryo models^86^) and additionally lacks tissues including the anterior neural, intermediate and lateral mesoderm; notochord; endoderm and non-neural ectoderm, among others. No human embryos were used in this research. All research with human ES cells was performed at the Francis Crick Institute under approval from the UK Stem Cell Bank Steering Committee (approval no. SCSC21-06). This work has been conducted in accordance with the 2021 ISSCR Guidelines.

### Mouse work

All experiments carried out on mice (*Mus musculus*) were conducted according to the UK Animal (Scientific Procedures) Act under licence no. PP6551133. The CD-1 animals were held within a Techniplast individually ventilated cage Green Line system, and the air movement in the cages was regulated by a Techniplast air management unit on negative pressure, 75 ACH/−20%, with all cages placed on automatic watering. The humidity and temperature were regulated at room level, set at code-of-practice standard levels of 20–24 °C and 55% (±10%) humidity. The light cycle was 7 a.m. to 7 p.m., including dawn and dusk settings. The animals were kept on Datesand Eco Pure Chips sawdust, Bed-r’Nest nesting and smart homes enrichment, with sterilized Teklad Global 18% Protein Rodent Diet (2018S).

Embryos were obtained from five pregnant adult female mice at E9.5 and were dissected from maternal and extraembryonic tissues. Before fixation, embryos were washed in PBS for a few minutes. Mouse embryos were fixed overnight in 4% paraformaldehyde (PFA; v/v; Alfa Aesar, 43368.9 M) in RNase-free PBS (Invitrogen, AM9625) at 4 °C, then dehydrated gradually into methanol by incubating them for 5 min in a series of increasing concentrations (0%, 25%, 50%, 75% and 100% by volume, respectively) in RNase-free PBS with 0.1% (v/v) Tween20 (Sigma-Aldrich, P1379) (PBST) on ice.

### Human stem cell lines

The cell lines used in this study include the human iPS cell lines HES7::Achilles^49^, SOX2::mEGFP (FCI-AICS-0074-026)^87^, ZO-1::mEGFP (FCI-AICS-0023)^87^, β-catenin::mEGFP (FCI-AICS-0058 cl.67)^87^, LaminB1::mTagRFP-T (FCI-AICS-0034 cl.62)^87^ and H2B::mEGFP (FCI-AICS-0061 cl.36)^87^; and the human ES cell lines RUES2-GLR^47^ and H9 SOX2::H2B-tdTomato/T::H2B-GFP dual reporter^88^ (H9-SBR). Cell lines engineered to report RA activity and associated control cell lines were derived from the parental WTC-11 cell line (Coriell Institute, GM25256) expanded at the Francis Crick Institute (FCI-GM25256). This research used Crick Human Embryo and Stem Cell Unit (now Human Biology) core facility expanded cell lines made possible through the Allen Cell Collection (NIGMS Human Genetic Cell Repository), available from the Coriell Institute for Medical Research (repository ID nos. AICS-0074-026, AICS-0023, AICS-0058, AICS-0034, AICS-0061 and GM25256). The HES7::Achilles iPS cell line was a gift from Olivier Pourquie. The H9-SBR ES cell line was a gift from Ting Zhou. The RUES2-GLR ES cell line was a gift from Ali Brivanlou. All cells were cultured in incubators at 37 °C and 5% CO_2_. Human PS cells were cultured routinely in either StemFit Basic 02 (Ajinomoto, discontinued in the European Union) supplemented with 80 ng ml^−1^ bFGF (PeproTech, 100-18B) or mTeSR 1 (STEMCELL Technologies, 85850) on 0.25 µg cm^−2^ recombinant Laminin iMatrix-511 silk E8-coated plates (AMSbio, AMS892 021). Cultures of 80–90% confluency were routinely passaged using Accutase (STEMCELL Technologies, 07922), and single cells were seeded with medium supplemented with 10 µM Y-27632 (ROCK inhibitor; Tocris Biosciences, 1254). Fresh medium was exchanged daily. Cells were regularly tested for mycoplasma contamination by PCR.

### Generation of RA activity reporting cell lines

Human cell lines genetically engineered to contain the RARE were established from FCI-GM25256 cells by transfection with the RARE/DR5 Lentiviral Dual-Reporter System (Fluorescence) (TRS-0043-2, LipExoGen Biotech). Two sets of cell lines were generated via lentiviral transfection, one reporting RA activity and the other as a negative control. In both systems, the lentiviral vector contains a dual fluorescent reporter system, containing (1) mCherry, constitutively expressed, and (2) eGFP, of which the expression is controlled by RA signalling. Specifically, eGFP expression is activated when RAR–RXR heterodimers bind to tandem direct-repeat-5 (DR5) response elements in the presence of the ligand ATRA. The control cell lines contain the eGFP sequence but lack the DR5 elements and therefore do not respond to ATRA.

FCI-GM25256 cells were transduced via lentiviral particles with the addition of polybrene 9 μg ml^−1^ (Sigma; TR-1003-G). Infection efficiency, as assessed by the proportion of mCherry+ cells, was evaluated 48–72 h later. A few days after infection, monoclonal and polyclonal cell lines for each construct were generated by fluorescence-activated cell sorting (BD FACSAria Fusion flow cytometer) sorting 1 or 100 mCherry+ cells, respectively, per well. Newly generated cell lines were expanded and cultured in mTeSR1 supplemented with 10 µg ml^−1^ blasticidin (Gibco, R21001) for selection. Functional validation of the reporter cell lines to 100 nM ATRA (Sigma-Aldrich, R2625) was evaluated after exposing the cells to ATRA for 24 h. Requests for use of this reporter line should be directed to the corresponding author.

### Human gastruloids

RUES2-GLR human ES cells grown routinely in NutriStem hPSC XF were first plated as single cells at a density of 5.2–6.7 × 10^3^ cells cm^−^^2^ on a layer of vitronectin (ThermoFisher, A14700; 5 μg ml^−1^, 0.5 μg cm^−^^2^) with 5 µM Y-27632 (Tocris Biosciences, 1254) for the first 24 h after plating. After further culture, they were pre-treated on the fourth day (96 h) with 4 µM CHIR99021 (Chiron; Tocris Biosciences, 4423) and 10 µM SB431542 (Stratech (Selleck Chemicals), S1067; TGFβ inhibitor) in NutriStem hPSC XF for 24 h. After this, they were dissociated in StemPro Accutase (Gibco Thermo Fisher Scientific, A1110501), and 700 cells were seeded into each well of an ultralow-adherence 96-well plate (Greiner Bio-One, 650970) in 50 μl Essential 6 medium (Gibco Thermo Fisher Scientific, A1516401) supplemented with 5 µM Y-27632 and 3 µM Chiron. The medium was replaced daily with fresh Essential 6. At 76 h, the structures were embedded in 20% (v/v) Corning Matrigel growth factor-reduced basement membrane matrix, phenol red free, lactate dehydrogenase-elevating virus free (Corning, 356231) or Corning Matrigel human ES cell-qualified matrix, lactate dehydrogenase-elevating virus free (Corning, 354277) in Essential 6 medium to a final protein concentration of 1.96 mg ml^−1^. The 20% (v/v) Matrigel solutions were prepared on ice with ice-cold Essential 6, before plating as 5-μl droplets onto tissue-culture treated plastic on ice. Human gastruloids were collected and transferred individually into separate 5-μl droplets with a micropipette. After transfer, droplets were left to gelate for 1 h in the incubator at 37 °C and 5% CO_2_, before covering gently with excess Essential 6 medium pre-warmed to 37 °C.

### Generating hTLS

The full protocol is available on https://www.protocols.io (ref. ^29^). In brief, once human PS cells were around 70–80% confluent, cells were dissociated in Accutase and seeded at a density dependent on the cell line (2–3.5 × 10^4^ cells per well; see protocol) per well of a laminin-coated six-well plate with 2 ml StemFit 02 with 80 ng ml^−1^ bFGF supplemented with 10 µM Y-27632. After 5 or 6 days, cultures were pre-treated in StemFit 02 with 20 ng ml^−1^ bFGF supplemented with CHIR99021 (Chiron; Tocris Biosciences, 4423). Alternatively, cultures maintained in mTeSR1 were pre-treated with N2B27 supplemented with CHIR99021. N2B27 medium was prepared from a 1:1 mix of Advanced DMEM/F12 (Gibco, 12634028) and Neurobasal medium (Gibco, 21103049) supplemented with N-2 Supplement (100×; Gibco, 17502048), B27 supplement (50×) without vitamin A (Gibco, 12587010) and 1% GlutaMAX supplement (v/v; Gibco, 35050061). The optimal concentration of Chiron for pre-treatment needed to be adjusted depending on the cell line and medium used but ranged between 2 and 5 µM. The concentrations for the lines used in this study are presented in Supplementary Table 1.

After 24 h of pre-treatment, cells were dissociated with Accutase. Cell numbers were determined using an automated cell counter (NuceloCounter NC-202, ChemoMetec), and 500 cells were seeded into one well of an ultralow-adherence 96-well plate (Greiner Bio-One, 650970) with 50 μl of either StemFit medium with 20 ng ml^−1^ bFGF or N2B27 (depending on the medium used for pre-treatment) and supplemented with 10 µM Y-27632, 10 µM SB431542 (TGFβ inhibitor) and a cell line-dependent concentration of Chiron (Supplementary Table 1). The plate was centrifuged at 160*g* at room temperature (RT) for 2 min to form a cell pellet at the bottom of the well. The following day, 150 μl fresh StemFit 02 or N2B27 was added to each well and exchanged again after 24 h with 150 μl medium. At 72 h after aggregation, 150 μl medium was removed from aggregates and they were embedded into medium containing 10% (v/v) growth factor-reduced Matrigel (Corning, 356231; protein concentration 7.3–8.8 mg ml^−1^) and 1 µM all-*trans* Retinal (Merck Sigma-Aldrich, R2500-100MG), unless stated otherwise. No further medium changes were required from this point.

### SEM

hTLSs, made from the LaminB1::mTagRFP-T line at 120 h after aggregation, were recovered from Matrigel using Cell Recovery Solution (Corning, 354253) for 30 min at 4 °C, washed twice with Dulbecco’s PBS without Mg^2+^ or Ca^2+^ (Gibco, 14190144) and fixed overnight in 4% PFA (v/v) in PBS at 4 °C.

They were then additionally fixed in 4% PFA/2.5% glutaraldehyde (v/v) in 0.1 M phosphate buffer (PB) for 1 h at RT, washed three times for 5 min in 1% bovine serum albumin (BSA, v/v) in 0.1 M PB, post-fixed in 1% reduced osmium (1% OsO_4_/1.5% K_3_Fe(CN)_6_; v/v) for 1 h at 4 °C, washed three times for 5 min in PB and then dehydrated with a graded ethanol series (70%, 90% and 2 × 100% by volume) for 20 min each at RT. The samples were then critical point dried (Leica EM CPD300 Critical Point Dryer), mounted on stubs, sputter coated with 5 nm of platinum (Quorum 150RS) and then imaged on the Quanta 250 FEG SEM (Thermo Fisher Scientific).

The SEM was operated between a chamber pressure of 4.0 and 4.38 Pa, with 30 μm aperture inserted using an accelerating voltage of 2.5 kV (Fig. 1e, top right), 3 kV (Fig. 1e, left) and 5 kV (Fig. 1e, middle and bottom right); a dwell time of 20 μs; and a working distance of 7.4 mm (Fig. 1e, bottom right), 7.6 mm (Fig. 1e, middle right), 9.2 mm (Fig. 1e, left) and 11.6 mm (Fig. 1e, top right). A 90° tilt sample holder was used for Fig. 1e (top right).

### Immunostaining

Pre-embedded structures were transferred into 1.5-ml tubes for fixation. Some structures were first recovered from Matrigel using Cell Recovery Solution for 30 min at 4 °C (Figs. 4c,e and 6a and Extended Data Figs. 7g (top), 8c and 10c,e,g), although we noticed some lack of morphological integrity upon exposure, so this was not used routinely. They were then washed with PBS twice, fixed overnight in 4% PFA (v/v) at 4 °C and then washed again three times for 10 min in PBS. Then, samples were washed three times for 10 min each in PBS with 0.2% Triton X-100 (v/v) and 3% BSA (w/v) (PBS-BT) at RT and blocked for 2 h in PBS-BT at 4 °C. Primary antibodies were diluted in blocking solution and incubated overnight at 4 °C, with gentle rocking. Following several washes in PBS-BT (2 × 5 min at RT, 3 × 10 min at 4 °C and 4 × 30 min at 4 °C), samples were incubated with Alex-Fluor-conjugated secondary antibodies with 1:500 Phalloidin CruzFluor 647 (Santa Cruz, sc-363797) or 1:800 Hoechst 33342 (Invitrogen, H3570) in PBS-BT with 10% normal donkey serum (v/v) overnight at 4 °C, with gentle rocking away from light. After additional washes (3 × 10 min in PBS-BT at RT, 2 × 5 min in PBS with 0.2% Triton X-100 (Merck, T8787) and 0.2% BSA (PBT) and 3 × 15 min in PBT at RT), samples were mounted in Vectashield (Vector Labs, H-1900-10) or RapiClear 1.52 (SunJin Lab, #RC152001:10 ml) onto either a Superfrost microscopy slide (Epredia, AA00008032E01MNZ10) or coverslip (VWR, 631-0138).

The antibodies used in this study were 1:200 rabbit anti-Brachyury (Abcam, ab209665); 1:500 mouse anti-MEOX1 (ThermoFisher, TA-804716); 1:200 goat anti-SOX2 (R&D Systems, AF2018-SP), 1:200 rat anti-SOX2 (Invitrogen, 14-9811-82), 1:400 rabbit anti-PAX6 (ThermoFisher, 42-6600) and 1:500 rabbit anti-ALDH1A2 (Sigma-Aldrich, HPA010022). The mouse anti-PAX7 monoclonal antibody (1:10) developed by A. Kawakami, Tokyo Institute of Technology, was obtained from the Developmental Studies Hybridoma Bank, created by the National Institute of Child Health and Human Development of the National Institutes of Health and maintained at the Department of Biology of the University of Iowa. All secondary antibodies were raised in donkey and diluted 1:500, and included Alexa Fluor 488-, 594- and 647-conjugated antibodies (Jackson ImmunoResearch). All primary antibodies were validated for sensitivity and specificity by the manufacturers, are reactive to human proteins and have been previously published.

### HCR

Custom probe sets (all probe set sizes, 20 pairs), hybridization chain reaction (HCR) amplifiers and buffers (hybridization, wash and amplification buffers) were purchased from Molecular Instruments. Pre-embedded structures were fixed in 4% PFA (v/v) in RNase-free PBS overnight, as described above. Samples were dehydrated gradually into methanol by incubating them for 5 min in a series of increasing concentrations (0%, 25%, 50%, 75% and 100% by volume, respectively) in PBST and stored at −20 °C. For the HCR, samples were rehydrated gradually by incubating them for 5 min in a series of decreasing concentrations of methanol (75%, 50%, 25% and 0% by volume, respectively) in PBST. Samples were washed once more in PBST, incubated for 5 min with hybridization buffer at RT and then for a further 30 min at 37 °C, alongside a combination of probes (8 nM final), in hybridization buffer. Samples were then incubated with the probe solution overnight at 37 °C. To remove excess probes, samples were washed with probe wash buffer four times for 15 min each at 37 °C, followed by a further three washes in 5× SSCT three times for 5 min at RT. Samples were pre-amplified in amplification buffer for 30 min at RT, gently rocking. Amplifier hairpins h1 and h2 were snap-cooled separately by first heating at 95 °C for 90 s and then cooling down at RT for 30 min in the dark. Hairpin amplifiers were diluted to 6 nM in amplification buffer, added to samples and then incubated overnight in the dark at RT. Next, samples were washed in 5 × SSCT four times for 5 min, then two times for 30 min, with a final wash in PBST for 2 min. Samples were counterstained 1:1000 with Hoechst 33342 overnight at 4 °C. The next day, samples were washed three times with PBST for 5 min and post-fixed with 4% PFA (v/v) in RNase-free PBS for 20 min. Thereafter, they were washed a further three times with PBST for 5 min at 4 °C with gentle rocking. Samples were mounted in Vectashield or Prolong Glass Antifade Mountant (Invitrogen, P36980) onto either a Superfrost microscopy slide or coverslip, then coverslipped. The HCR probe targets and the associated amplifiers used are presented in Supplementary Table 2.

For HCR of mouse embryos, samples were rehydrated gradually by incubating them for 5 min in a series of decreasing concentrations of methanol (75%, 50%, 25% and 0% by volume, respectively) in PBST. Embryos were bleached in 6% H_2_O_2_ (v/v) in PBS for 15 min and were washed three times for 5 min each in PBST at RT. Samples were treated with 10 μg ml^−1^ Proteinase K (Roche, 3115879001) for 10 min (no rocking) and then washed twice for 5 min in PBST (no rocking). Afterwards, embryos were post-fixed for 20 min with 4% PFA (v/v) in RNase-free PBS at RT, gently rocking, and then washed three times for 5 min with PBST. To equilibrate embryos, samples were washed in 1:1 pre-warmed hybridization buffer and PBST for 5 min. Embryos were washed with pre-warmed hybridization buffer for 5 min before following the HCR protocol as described above.

### Imaging

Full details of imaging hardware and settings are provided in Supplementary Table 3.

#### Confocal imaging

Confocal imaging was performed using a Zeiss Invert880 with Airyscan 2014 or Zeiss Invert880 NLO OPO, using a Zeiss Pan-Apochromat 20×/0.8 objective. *Z*-stacks were obtained with 5-µm steps, averaging each slice four times, unless stated otherwise. Any tiled images were acquired with a 10% overlap. Spectral unmixing was used for imaging HCR samples, unless stated otherwise (track method used for Figs. 1d, 2f, 3i,j and 5b,c and Extended Data Figs. 1h and 9a). In brief, spectra were set up for all HCR amplifiers by imaging droplets of snap-cooled mixed-hairpin pairs using lambda mode, and then areas were selected to save each spectrum to a spectral database. HCR images were recorded using lambda mode and linear unmixing was run with saved spectra of amplifiers, nuclear signal and autofluorescence spectra of the hTLS. Imaging data were acquired with Zeiss ZEN Black 2.3 SP1 software. Confocal images are presented either as optical slices or maximum intensity projections, generated using FIJI (2.14.0/1.54f) or Imaris (10.1.1, Oxford Instruments), unless stated otherwise. For Fig. 5g, *XY* images and *YX* optical slices were taken of hTLSs imaged in Fig. 5e using average mode in Imaris.

#### Live imaging

Brightfield, widefield or confocal spinning disk images on live samples were obtained on an ImageXpress Confocal HT.ai system with Nikon 10× S Plan Apo Lambda objective. Time-lapse imaging was acquired with intervals between 20 and 30 min under environmental control at 37 °C and 5% CO_2_. Data were acquired using MetaXpress (6.7.1.157) software.

#### Lightsheet imaging

After post-fixation following the HCR protocol, mouse embryos for lightsheet imaging were embedded in a cryomold in warm 1% ultralow melting agarose (Sigma, A5030) and placed on ice to polymerize. Samples were then prepared for clearing with a 1:2 solution of benzyl alcohol (Sigma, 24122) and benzyl benzoate (ThermoFisher, 105860010) (BABB). All steps were carried out in a glass vial, which was covered with foil. Mouse embryos were dehydrated gradually into methanol by incubating them for 1 h at RT with gentle rocking in a series of increasing concentrations (50%, 70%, 80%, 90%, and twice in 100% by volume, respectively) in deionized water. Embryos were then incubated in a solution of 1:1 100% methanol and BABB for 1 h at RT with gentle rocking. Samples were then transferred into a 100% solution of BABB and left overnight. The next day, BABB was removed and the samples were washed in ethyl cinnamate (ECi; Sigma, 112372) for 5 min before transferring samples into fresh ECi for storage. Mouse embryos were imaged using Miltenyi-Lavision UM II with ECi as the imaging solution. *Z*-stacks were obtained with 1.88–4 µm steps with dynamic focus. Imaging data were collected with Imspector (7.5.4) software. Lightsheet images and videos were generated with Imaris.

### Image analysis

#### NMP colocalization analysis (whole volume segmentation)

Confocal images of hTLS stained with Hoechst, TBXT and SOX2 were processed through Imaris. Surfaces of whole hTLSs were generated from the Hoechst, TBXT and SOX2 channels. A new channel of the colocalized region was generated using the colocalization package in Imaris, and then a new surface was generated from this channel.

#### NMP quantification

Confocal images of hTLSs stained with Hoechst, TBXT and SOX2 were processed through Imaris. Spots of nuclei were generated from the Hoechst channel and then subset to those that co-expressed TBXT and SOX2 above set thresholds that were the same across all conditions (TBXT >7,927; SOX2 >7,625). The numbers of NMP spots were then normalized to the total number of spots (nuclei).

#### Polarization of gene expression

Confocal images were imported into Imaris, and the Hoechst channel was used to create a manual surface rendering of the whole hTLS, the individual somites and the neural tube. New channels were created for fluorescent HCR signal within each structure, and this was then used to generate surfaces corresponding to positive signals within each somite.

#### Quantification of DLL1+ cells

Confocal images were imported into Imaris before using either the SOX2 channel automatically or the Hoechst channel manually to create a surface rendering of the neural tube. DLL1 expression was masked within this surface, and spots were created corresponding to the DLL1 signal within the neural tube.

#### Morphometric analysis

The pixel classification tool Ilastik (1.4.1rc2)^89^ was used to label hTLS (24–144 h) in transmitted light images to generate probability maps, distinguishing the hTLS from background. CellProfiler (4.2.8)^90^ was used to obtain basic morphometry through the generation and measurement of objects using these probability maps. Objects touching the edge of the image and those below a pixel threshold were automatically discarded. Images were then manually checked, and sample images containing fibres and debris were discarded. The medial length was obtained through CellProfiler via ImageJ^91^: objects were skeletonized, and the largest, shortest branch length was selected. Quantification of somite number was measured through manual counting of segmented structures on 96–144 h hTLSs.

#### Oscillation quantification

HES7-Achilles samples were imaged on the ImageXpress Confocal HT.ai system from 100 to 115.75 h in the hTLS protocol. Initial image analysis was performed in FIJI^92^, where regions of interest for HES7 oscillations and background were selected. Integrated density, mean pixel intensity and area were measured and inputted into the following formula: corrected total fluorescence = integrated density – (area of selected cell × mean fluorescence of background readings). De-trending was then performed in terminal on MacOS, and peaks and valleys were calculated in Microsoft Excel (16.101.2) and plotted in RStudio (2023.06.0+421).

### Signalling manipulation

For RA signalling manipulation of hTLSs, retinal was replaced with either dimethylsulfoxide or ATRA (100 nM) or treated with BMS493 (2.5 µM; Tocris 3509) at 72 h during embedding. In addition, for 96-h RA inhibition of hTLS, BMS493 was added to 20 µl medium containing 10% growth factor-reduced Matrigel (v/v) and 1 µM all-*trans* retinal and gently added to each well for a final concentration of 2.5 µM.

For SHH signalling activation of hTLS models, SAG (Tocris, 4366) was added to 20 µl medium containing 10% growth factor-reduced Matrigel (v/v) and 1 µM all-*trans* retinal and gently added to each well for the following final concentrations: 100 nM, 200 nM, 300 nM, 400 nM and 500 nM at 96 h. For RA inhibition of Shh signalling-activated hTLS, BMS493 was added to 20 µl medium containing 10% growth factor-reduced Matrigel (v/v) and 1 µM all-*trans* retinal for a final concentration of 2.5 µM BMS493 with final SAG concentrations of 100 nM, 200 nM, 300 nM, 400 nM and 500 nM at 96 h.

For Wnt signalling manipulation of 96 h hTLS, Wnt inhibitors, IWP 2 (PORCN inhibitor; Tocris, 3533) and XAV-939 (Selleck Chemicals, S1180) and Wnt activators, WNT5A (Bio-techne, 645-WN) and WNT5B (Bio-techne, 7347-WN), were added to 20 µl medium containing 10% growth factor-reduced Matrigel (v/v) and 1 µM all-*trans* retinal for final concentrations of 5 µM, 5 µM, 100 ng ml^−1^ and 100 ng ml^−1^, respectively.

### Single-cell RNA sequencing

Samples from each timepoint were collected from 96-well plates and pooled together. TrypLE Select was added to the samples and they were incubated at 37 °C for 15–20 min. Samples were gently pipetted up and down until the hTLSs were dissociated into single cells. The cells were then spun in the centrifuge at 170*g* for 4 min and resuspended in PBS^−/−^ + 0.04% BSA (v/v) and kept on ice until further processing. The concentration and viability of the single-cell suspension was measured using acridine orange and propidium iodide with a Luna-FX7 Automatic Cell Counter. A total of 13,200 cells were loaded onto a chromium chip and partitioned in nanolitre scale droplets using the Chromium Controller and Chromium Next GEM Single Cell Reagents (where GEM is Gel Bead-in Emulsion; CG000315 Chromium Single Cell 3′ Reagent Kits User Guide (v3.1, Dual Index)). Within each droplet, the cells were lysed and the RNA was reverse transcribed. All of the resulting complementary DNA within a droplet shared the same cell barcode. Illumina-compatible libraries were generated from the cDNA using Chromium Next GEM Single Cell library reagents in accordance with the manufacturer’s instructions (10x Genomics, CG000315 Chromium Single Cell 3′ Reagent Kits User Guide (v3.1, Dual Index)). Final libraries were quality controlled using the Agilent TapeStation and sequenced using the Illumina NovaSeq 6000. The sequencing read configuration was 28–10–10–90.

### Bioinformatic analysis

FASTQ files were aligned to the hg38 transcriptome, and count matrices were generated, filtering for GEM cell barcodes (excluding GEMs with free-floating mRNA from lysed or dead cells) using CellRanger (version 6.0.1). Count matrices were imported into R (version 4.1.0) and processed using the Seurat library (version 4.4.0) following the standard pipeline^93^. Low-quality cells were removed, with cells kept for further analysis if they met the following criteria: the mitochondrial content was within three standard deviations from the median, more than 500 genes were detected and more than 1,000 RNA molecules were detected. DoubletFinder was used to identify doublets, assuming a theoretical doublet rate of 7.5%, which were removed from subsequent analysis^94^. In total, 18,405 cells were sequenced, of which 17,661 cells passed the quality control and 15,808 passed subsequent doublet filtration for downstream analysis (5,979 cells from the 72 h sample, 3,669 cells from the 96 h sample and 6,160 cells from the 120 h sample).

All samples were integrated using the reciprocal principal component analysis (RPCA) method, implemented by Seurat’s functions FindIntegrationAnchors() and IntegrateData(), using the top 3,000 variable features and the first 25 principal components as determined using the ‘maxLikGlobalDimEst’ function from the intrinsicDimension package (version 1.2.0). Effects of cell cycle heterogeneity was calculated using cell cycle phase scores on the basis of canonical markers and regressed out of the data using ScaleData. Eight clusters were identified at a resolution of 0.5.

For RNA velocity analysis, loom files containing spliced and unspliced matrices were calculated, from BAM files generated by CellRanger, using the package velocyto (version 0.17.8) and Python 3.6.4. The matrices were used as input to scVelo (version 0.2.4) to calculate the RNA velocity values for each gene of each cell using the package loompy (version 3.0.6) and scanpy (version 1.8.2). scVelo was used in the dynamical mode with default settings. The resulting RNA velocity vector was overlaid onto the UMAP space by translating the RNA velocities into likely cell transitions using cosine correlation to compute the probabilities of one cell transitioning into another cell.

Lineages for trajectory analyses were identified separately for the somite and neural fates with the NMP cluster as the starting population using Monocle3 (version 1.3.0)^95^. Differentially expressed genes along each of the lineages were identified with the ‘graph_test’ function using the Morans *I* test. The top 100 genes were used to draw smoothed heat maps using Tradeseq (version 1.7.07)^96^ and ComplexHeatmap (version 2.2.0)^97^.

Cell-to-cell crosstalk was inferred using the R library CellChat (version 2.1.2)^74^ and the CellChat human database. Clusters designated NP1, NP2, NP3 and neuronal were merged to create one cluster. Only the somites and the merged neuronal clusters were considered for Cellchat analysis. The CellChat analysis was performed as outlined in the CellChat vignette, with the ‘population.size’ parameter set to ’true’ when computing the communication probability between clusters. Significant interactions were filtered on inter-cluster and intra-cluster interactions were removed.

Published datasets were reprocessed using Seurat^93^ (version 4.4.0) from either the counts matrix of a Seurat object or downloaded outputs of CellRanger from the Gene Expression Omnibus (GEO) repository of the National Center for Biotechnology Information (NCBI).

Four samples (comprising hAxioloid_48h, hAxioloid_72h, 96h_MG_RAL and 120h_MG_RAL from the axioloid dataset (GSE199576)^15^) were downloaded as count matrices and processed using Seurat version 4.4.0. Samples were integrated using methods highlighted in the authors’ publication and cells assigned clusters according to published cell metadata. Cells from this dataset were projected onto our integrated dataset using the functions ‘FindTransferAnchors’ (using the first 30 dimensions of the principle component analysis space) and ‘MapQuery’. To determine the similarity between axioloid clusters and hTLS-integrated clusters, a two-tailed Spearman’s rank correlation was calculated on the aggregated cell expression using the mean gene expression per cluster and per sample, after filtering on the basis of common genes between datasets.

The full human embryo dataset (GSE157329)^38^ was downloaded and processed using scripts provided by the authors (https://github.com/zhirongbaolab/heoa) with Seurat 4.4.0. Cells were annotated using published annotation. Predicted cell type cluster and stage annotation scores were filtered using a threshold of >0.5.

The human neural tube dataset^71^ was used to look for similarity of clusters within our integrated neuronal clusters (labelled NP1, NP2, NP3 and neuronal). A two-tailed Spearman’s rank correlation test was used on aggregated cell expression per cluster using common genes between both datasets.

Single-cell datasets from refs. ^15,21,22,38^ and our hTLS data were integrated using harmony (https://github.com/immunogenomics/harmony) to account for experimental batch effects with the following parameters (group.by.vars = experiment; reduction.used = pca, nclust = 100). The normalized expression for the integrated multi-experiment dataset was aggregated per cluster. To look for similarities between clusters, a Spearman’s correlation coefficient was calculated using the normalized aggregated expression for genes, which was identified as the union of the top 50 markers per cluster per dataset. The Spearman’s correlations are shown as a tile plot, where the rows and columns are hierarchically clustered, using the ‘ward.d2’ method.

### Statistics and reproducibility

No statistical method was used to pre-determine the sample size, but our sample sizes are similar to those reported in previous publications^15^. Statistical testing included tests of normality to guide the use of appropriate statistical methods. Data were excluded from morphometric analyses where images were of empty wells or debris, where structures were partially out of the field of view, where pixel values fell beneath a threshold for object detection or where manual validation identified poor segmentation. In bioinformatic analyses, low-quality cells or doublets (identified by DoubletFinder with a theoretical rate of 7.5%) were removed from downstream analysis. Cells were only retained if they met the following criteria: the mitochondrial content was within three standard deviations from the median, more than 500 genes were detected and more than 1,000 RNA molecules were detected. The experiments were not randomized. The investigators were not blinded to allocation during the experiments and outcome assessment. Data collection and analyses were not performed blinded to the conditions of the experiments.

### Reporting summary

Further information on research design is available in the Nature Portfolio Reporting Summary linked to this article.

## Online content

Any methods, additional references, Nature Portfolio reporting summaries, source data, extended data, supplementary information, acknowledgements, peer review information; details of author contributions and competing interests; and statements of data and code availability are available at 10.1038/s41556-025-01813-8.

## Supplementary information

Supplementary Information Supplementary Fig. 1 and Table legends 1–3.

Reporting Summary

Supplementary Tables Supplementary Table 1. Chiron concentrations at pre-treatment and aggregation by cell line. Supplementary Table 2. HCR probe list. Supplementary Table 3. Light microscopy reporting table.

Supplementary Video 1 **Supplementary Video 1: ZO-1::mEGFP expression in hTLS**. Live-cell imaging of ZO-1::mEGFP human iPSC cell line, showing widefield and ZO-1::mEGFP expression in hTLS from 72.5-96 h (*n* = 6). Scale bars, 100 μm.

Supplementary Video 2 **Supplementary Video 2: HES7 oscillatory expression in hTLS**. Live-cell imaging of HES7::Achillies human iPSC cell line, showing HES7 expression in hTLS from 100-121 h (*n* = 33). Scale bars, 100 μm.

Supplementary Video 3 **Supplementary Video 3:** ***ALDH1A2*** **expression in somites of mouse embryos**. Localised *ALDH1A2* expression found in the medial somites relative to the neural tube (*n* = 4). Scale bars, 200 μm.

Supplementary Data 1 Source data for Supplementary Fig. 1.

## Source data

Source Data Figs. 1–4 and 6 and Extended Data Figs. 1–8 and 10. Statistical source data.

## Data Availability

Sequencing data that support the findings of this study have been deposited in the Gene Expression Omnibus (GEO) repository (GSE268451). Previously published single cell transcriptomic data that were re-analysed here are available under accession codes GSE157329, GSE199576, GSE220563 and GSE208369. All other data supporting the findings of this study are available from the corresponding author on reasonable request. Source data are provided with this paper.

## References

[1] Rekler, D. & Kalcheim, C. Completion of neural crest cell production and emigration is regulated by retinoic-acid-dependent inhibition of BMP signaling. *eLife* **11**, e72723 (2022). 35394423 10.7554/eLife.72723 PMC8993216

[2] Pourquié, O. et al. Lateral and axial signals involved in avian somite patterning: a role for BMP4. *Cell* **84**, 461–471 (1996). 8608600 10.1016/s0092-8674(00)81291-x

[3] Rong, P. M., Teillet, M. A., Ziller, C. & Le Douarin, N. M. The neural tube/notochord complex is necessary for vertebral but not limb and body wall striated muscle differentiation. *Development* **115**, 657–672 (1992). 1425345 10.1242/dev.115.3.657

[4] Wang, G. et al. Misexpression of BRE gene in the developing chick neural tube affects neurulation and somitogenesis. *Mol. Biol. Cell* **26**, 978–992 (2015). 25568339 10.1091/mbc.E14-06-1144 PMC4342032

[5] Buffinger, N. & Stockdale, F. E. Myogenic specification in somites: induction by axial structures. *Development* **120**, 1443–1452 (1994). 8050355 10.1242/dev.120.6.1443

[6] Münsterberg, A. E. & Lassar, A. B. Combinatorial signals from the neural tube, floor plate and notochord induce myogenic bHLH gene expression in the somite. *Development* **121**, 651–660 (1995). 7720573 10.1242/dev.121.3.651

[7] Lee, K. K. et al. Desmin transgene expression in mouse somites requires the presence of the neural tube. *Int. J. Dev. Biol.* **39**, 469–475 (1995). 7577437

[8] del Corral, R. D. et al. Opposing FGF and retinoid pathways control ventral neural pattern, neuronal differentiation, and segmentation during body axis extension. *Neuron* **40**, 65–79 (2003). 14527434 10.1016/s0896-6273(03)00565-8

[9] Pera, M. F. Human embryo research and the 14-day rule. *Development* **144**, 1923 (2017). 28559237 10.1242/dev.151191

[10] Rugg-Gunn, P. J., Moris, N. & Tam, P. P. L. Technical challenges of studying early human development. *Development*10.1242/dev.201797 (2023). 10.1242/dev.201797 PMC10281548 37260362

[11] Moris, N., Martinez Arias, A. & Steventon, B. Experimental embryology of gastrulation: pluripotent stem cells as a new model system. *Curr. Opin. Genet. Dev.* **64**, 78–83 (2020). 32663757 10.1016/j.gde.2020.05.031

[12] Moris, N. et al. An in vitro model of early anteroposterior organization during human development. *Nature* **582**, 410–415 (2020). 32528178 10.1038/s41586-020-2383-9

[13] van den Brink, S. C. et al. Single-cell and spatial transcriptomics reveal somitogenesis in gastruloids. *Nature* **582**, 405–409 (2020). 32076263 10.1038/s41586-020-2024-3

[14] Veenvliet, J. V. et al. Mouse embryonic stem cells self-organize into trunk-like structures with neural tube and somites. *Science* **370**, eaba4937 (2020). 33303587 10.1126/science.aba4937

[15] Yamanaka, Y. et al. Reconstituting human somitogenesis in vitro. *Nature* **614**, 509–520 (2023). 36543322 10.1038/s41586-022-05649-2

[16] Sanaki-Matsumiya, M. et al. Periodic formation of epithelial somites from human pluripotent stem cells. *Nat. Commun.* **13**, 2325 (2022). 35484123 10.1038/s41467-022-29967-1 PMC9050736

[17] Miao, Y. et al. Reconstruction and deconstruction of human somitogenesis in vitro. *Nature* **614**, 500–508 (2023). 36543321 10.1038/s41586-022-05655-4 PMC10018515

[18] Libby, A. R. G. et al. Axial elongation of caudalized human organoids mimics aspects of neural tube development. *Development* **148**, 10.1242/dev.198275 (2021). 10.1242/dev.198275 PMC8254868 34142711

[19] Karzbrun, E. et al. Human neural tube morphogenesis in vitro by geometric constraints. *Nature* **599**, 268–272 (2021). 34707290 10.1038/s41586-021-04026-9 PMC8828633

[20] Gribaudo, S. et al. Self-organizing models of human trunk organogenesis recapitulate spinal cord and spine co-morphogenesis. *Nat. Biotechnol.*10.1038/s41587-023-01956-9 (2023). 10.1038/s41587-023-01956-9 37709912

[21] Hamazaki, N. et al. Retinoic acid induces human gastruloids with posterior embryo-like structures. *Nat. Cell Biol.* **26**, 1790–1803 (2024). 39164488 10.1038/s41556-024-01487-8 PMC11469962

[22] Yaman, Y. I. & Ramanathan, S. Controlling human organoid symmetry breaking reveals signaling gradients drive segmentation clock waves. *Cell* **186**, 513–527.e519 (2023). 36657441 10.1016/j.cell.2022.12.042 PMC10025047

[23] Urzi, A. et al. Efficient generation of a self-organizing neuromuscular junction model from human pluripotent stem cells. *Nat. Commun.* **14**, 8043 (2023). 38114482 10.1038/s41467-023-43781-3 PMC10730704

[24] Faustino Martins, J. M. et al. Self-Organizing 3D Human Trunk Neuromuscular Organoids. *Cell Stem Cell* **26**, 172–186.e176 (2020). 31956040 10.1016/j.stem.2019.12.007

[25] Rito, T., Libby, A. R. G., Demuth, M. & Briscoe, J. Timely TGFβ signalling inhibition induces notochord. *Nature* **637**, 673–682 (2025). 10.1038/s41586-024-08332-w PMC11735409 39695233

[26] Kenny-Mobbs, T. & Thorogood, P. Autonomy of differentiation in avian brachial somites and the influence of adjacent tissues. *Development* **100**, 449–462 (1987). 3308405 10.1242/dev.100.3.449

[27] del Corral, R. D., Breitkreuz, D. N. & Storey, K. G. Onset of neuronal differentiation is regulated by paraxial mesoderm and requires attenuation of FGF signalling. *Development* **129**, 1681–1691 (2002). 11923204 10.1242/dev.129.7.1681

[28] Martin, B. L. & Kimelman, D. Canonical Wnt signaling dynamically controls multiple stem cell fate decisions during vertebrate body formation. *Dev. Cell* **22**, 223–232 (2012). 22264734 10.1016/j.devcel.2011.11.001 PMC3465166

[29] Makwana, K. et al. Generation of human trunk-like structures (hTLS) from human pluripotent stem cells. *Protocols.io*10.17504/protocols.io.e6nvw4d59lmk/v1 (2025).

[30] Thomson, L., Muresan, L. & Steventon, B. The zebrafish presomitic mesoderm elongates through compaction–extension. *Cells Dev.* **168**, 203748 (2021). 34597846 10.1016/j.cdev.2021.203748 PMC7612712

[31] Mongera, A. et al. A fluid-to-solid jamming transition underlies vertebrate body axis elongation. *Nature* **561**, 401–405 (2018). 30185907 10.1038/s41586-018-0479-2 PMC6148385

[32] Nikolopoulou, E., Galea, G. L., Rolo, A., Greene, N. D. & Copp, A. J. Neural tube closure: cellular, molecular and biomechanical mechanisms. *Development* **144**, 552–566 (2017). 28196803 10.1242/dev.145904 PMC5325323

[33] Greene, N. D. & Copp, A. J. Neural tube defects. *Annu. Rev. Neurosci.* **37**, 221–242 (2014). 25032496 10.1146/annurev-neuro-062012-170354 PMC4486472

[34] Chloe, S. et al. Spinal neural tube formation and tail development in human embryos. *eLife* **12**, RP88584 (2024). 39636098 10.7554/eLife.88584 PMC11620743

[35] Catala, M. Overview of secondary neurulation. *J. Korean Neurosurg. Soc.* **64**, 346–358 (2021). 33906344 10.3340/jkns.2020.0362 PMC8128529

[36] Mayeuf-Louchart, A. et al. Notch regulation of myogenic versus endothelial fates of cells that migrate from the somite to the limb. *Proc. Natl Acad. Sci. USA* **111**, 8844–8849 (2014). 24927569 10.1073/pnas.1407606111 PMC4066530

[37] Sahai-Hernandez, P. et al. Dermomyotome-derived endothelial cells migrate to the dorsal aorta to support hematopoietic stem cell emergence. *eLife* **12**, e58300 (2023). 37695317 10.7554/eLife.58300 PMC10495111

[38] Xu, Y. et al. A single-cell transcriptome atlas profiles early organogenesis in human embryos. *Nat. Cell Biol.* **25**, 604–615 (2023). 36928764 10.1038/s41556-023-01108-w

[39] Deschamps, J. & Duboule, D. Embryonic timing, axial stem cells, chromatin dynamics, and the Hox clock. *Genes Dev.* **31**, 1406–1416 (2017). 28860158 10.1101/gad.303123.117 PMC5588924

[40] Hunt, P. & Krumlauf, R. Hox codes and positional specification in vertebrate embryonic axes. *Annu. Rev. Cell Dev. Biol.* **8**, 227–256 (1992). 10.1146/annurev.cb.08.110192.001303 1362074

[41] Young, T. et al. *Cdx* and *Hox* genes differentially regulate posterior axial growth in mammalian embryos. *Dev. Cell* **17**, 516–526 (2009). 19853565 10.1016/j.devcel.2009.08.010

[42] Aires, R. et al. Tail bud progenitor activity relies on a network comprising *Gdf11*, *Lin28*, and *Hox13* genes. *Dev. Cell* **48**, 383–395 (2019). 30661984 10.1016/j.devcel.2018.12.004

[43] Cambray, N. & Wilson, V. Axial progenitors with extensive potency are localised to the mouse chordoneural hinge. *Development* **129**, 4855–4866 (2002). 12361976 10.1242/dev.129.20.4855

[44] Henrique, D., Abranches, E., Verrier, L. & Storey, K. G. Neuromesodermal progenitors and the making of the spinal cord. *Development* **142**, 2864–2875 (2015). 26329597 10.1242/dev.119768 PMC4958456

[45] Cambray, N. & Wilson, V. Two distinct sources for a population of maturing axial progenitors. *Development* **134**, 2829–2840 (2007). 17611225 10.1242/dev.02877

[46] Wymeersch, F. J. et al. Position-dependent plasticity of distinct progenitor types in the primitive streak. *eLife* **5**, e10042 (2016). 26780186 10.7554/eLife.10042 PMC4798969

[47] Martyn, I., Kanno, T. Y., Ruzo, A., Siggia, E. D. & Brivanlou, A. H. Self-organization of a human organizer by combined Wnt and nodal signalling. *Nature* **558**, 132–135 (2018). 29795348 10.1038/s41586-018-0150-y PMC6077985

[48] Pourquié, O. The segmentation clock: converting embryonic time into spatial pattern. *Science* **301**, 328–330 (2003). 12869750 10.1126/science.1085887

[49] Diaz-Cuadros, M. et al. In vitro characterization of the human segmentation clock. *Nature* **580**, 113–118 (2020). 31915384 10.1038/s41586-019-1885-9 PMC7336868

[50] Bessho, Y., Hirata, H., Masamizu, Y. & Kageyama, R. Periodic repression by the bHLH factor Hes7 is an essential mechanism for the somite segmentation clock. *Genes Dev.* **17**, 1451–1456 (2003). 12783854 10.1101/gad.1092303 PMC196074

[51] Bessho, Y. et al. Dynamic expression and essential functions of Hes7 in somite segmentation. *Genes Dev.* **15**, 2642–2647 (2001). 11641270 10.1101/gad.930601 PMC312810

[52] Schröter, C. et al. Dynamics of zebrafish somitogenesis. *Dev. Dyn.* **237**, 545–553 (2008). 18265021 10.1002/dvdy.21458

[53] Tenin, G. et al. The chick somitogenesis oscillator is arrested before all paraxial mesoderm is segmented into somites. *BMC Dev. Biol.* **10**, 24 (2010). 20184730 10.1186/1471-213X-10-24 PMC2836991

[54] Wahl, M. B., Deng, C., Lewandoski, M. & Pourquié, O. FGF signaling acts upstream of the NOTCH and WNT signaling pathways to control segmentation clock oscillations in mouse somitogenesis. *Development* **134**, 4033–4041 (2007). 17965051 10.1242/dev.009167

[55] Anderson, M. J., Magidson, V., Kageyama, R. & Lewandoski, M. Fgf4 maintains Hes7 levels critical for normal somite segmentation clock function. *eLife* **9**, e55608 (2020). 33210601 10.7554/eLife.55608 PMC7717904

[56] Maruoka, Y. et al. Comparison of the expression of three highly related genes, *Fgf8*, *Fgf17* and *Fgf18*, in the mouse embryo. *Mech. Dev.* **74**, 175–177 (1998). 9651520 10.1016/s0925-4773(98)00061-6

[57] Yamaguchi, T. P., Conlon, R. A. & Rossant, J. Expression of the fibroblast growth factor receptor FGFR-1/flg during gastrulation and segmentation in the mouse embryo. *Dev. Biol.* **152**, 75–88 (1992). 1321062 10.1016/0012-1606(92)90157-c

[58] Takada, S. et al. Wnt-3a regulates somite and tailbud formation in the mouse embryo. *Genes Dev.* **8**, 174–189 (1994). 8299937 10.1101/gad.8.2.174

[59] Aulehla, A. et al. Wnt3a plays a major role in the segmentation clock controlling somitogenesis. *Dev. Cell* **4**, 395–406 (2003). 12636920 10.1016/s1534-5807(03)00055-8

[60] Yu, H. M., Liu, B., Costantini, F. & Hsu, W. Impaired neural development caused by inducible expression of axin in transgenic mice. *Mech. Dev.* **124**, 146–156 (2007). 17123792 10.1016/j.mod.2006.10.002 PMC1847614

[61] Jho, E. H. et al. Wnt/beta-catenin/Tcf signaling induces the transcription of Axin2, a negative regulator of the signaling pathway. *Mol. Cell. Biol.* **22**, 1172–1183 (2002). 11809808 10.1128/MCB.22.4.1172-1183.2002 PMC134648

[62] Chitnis, A., Henrique, D., Lewis, J., Ish-Horowicz, D. & Kintner, C. Primary neurogenesis in *Xenopus* embryos regulated by a homologue of the *Drosophila* neurogenic gene *Delta*. *Nature* **375**, 761–766 (1995). 7596407 10.1038/375761a0

[63] Appel, B., Givan, L. A. & Eisen, J. S. Delta-Notch signaling and lateral inhibition in zebrafish spinal cord development. *BMC Dev. Biol.* **1**, 13 (2001). 11495630 10.1186/1471-213X-1-13 PMC37243

[64] Okigawa, S. et al. Different combinations of Notch ligands and receptors regulate V2 interneuron progenitor proliferation and V2a/V2b cell fate determination. *Dev. Biol.* **391**, 196–206 (2014). 24768892 10.1016/j.ydbio.2014.04.011

[65] Wahi, K., Bochter, M. S. & Cole, S. E. The many roles of Notch signaling during vertebrate somitogenesis. *Semin. Cell Dev. Biol.* **49**, 68–75 (2016). 25483003 10.1016/j.semcdb.2014.11.010

[66] Maden, M., Sonneveld, E., van der Saag, P. T. & Gale, E. The distribution of endogenous retinoic acid in the chick embryo: implications for developmental mechanisms. *Development* **125**, 4133–4144 (1998). 9753668 10.1242/dev.125.21.4133

[67] Swindell, E. C. et al. Complementary domains of retinoic acid production and degradation in the early chick embryo. *Dev. Biol.* **216**, 282–296 (1999). 10588879 10.1006/dbio.1999.9487

[68] Niederreither, K., McCaffery, P., Dräger, U. C., Chambon, P. & Dollé, P. Restricted expression and retinoic acid-induced downregulation of the retinaldehyde dehydrogenase type 2 (RALDH-2) gene during mouse development. *Mech. Dev.* **62**, 67–78 (1997). 9106168 10.1016/s0925-4773(96)00653-3

[69] Wilson, L. & Maden, M. The mechanisms of dorsoventral patterning in the vertebrate neural tube. *Dev. Biol.* **282**, 1–13 (2005). 15936325 10.1016/j.ydbio.2005.02.027

[70] Sagner, A. & Briscoe, J. Establishing neuronal diversity in the spinal cord: a time and a place. *Development*10.1242/dev.182154 (2019). 10.1242/dev.182154 31767567

[71] Rayon, T., Maizels, R. J., Barrington, C. & Briscoe, J. Single-cell transcriptome profiling of the human developing spinal cord reveals a conserved genetic programme with human-specific features. *Development*10.1242/dev.199711 (2021). 10.1242/dev.199711 PMC8353162 34351410

[72] Chen, J. K., Taipale, J., Cooper, M. K. & Beachy, P. A. Inhibition of Hedgehog signaling by direct binding of cyclopamine to Smoothened. *Genes Dev.* **16**, 2743–2748 (2002). 12414725 10.1101/gad.1025302 PMC187469

[73] Ribes, V. & Briscoe, J. Establishing and interpreting graded Sonic Hedgehog signaling during vertebrate neural tube patterning: the role of negative feedback. *Cold Spring Harb. Perspect. Biol.* **1**, a002014 (2009). 20066087 10.1101/cshperspect.a002014 PMC2742090

[74] Jin, S. et al. Inference and analysis of cell–cell communication using CellChat. *Nat. Commun.* **12**, 1088 (2021). 33597522 10.1038/s41467-021-21246-9 PMC7889871

[75] Molotkova, N., Molotkov, A., Sirbu, I. O. & Duester, G. Requirement of mesodermal retinoic acid generated by Raldh2 for posterior neural transformation. *Mech. Dev.* **122**, 145–155 (2005). 15652703 10.1016/j.mod.2004.10.008 PMC2826194

[76] Diez del Corral, R. & Morales, A. V. Retinoic acid signaling during early spinal cord development. *J. Dev. Biol.* **2**, 174–197 (2014).

[77] Wilson, L., Gale, E., Chambers, D. & Maden, M. Retinoic acid and the control of dorsoventral patterning in the avian spinal cord. *Dev. Biol.* **269**, 433–446 (2004). 15110711 10.1016/j.ydbio.2004.01.034

[78] Germain, P. et al. Differential action on coregulator interaction defines inverse retinoid agonists and neutral antagonists. *Chem. Biol.* **16**, 479–489 (2009). 19477412 10.1016/j.chembiol.2009.03.008

[79] Niederreither, K., Subbarayan, V., Dollé, P. & Chambon, P. Embryonic retinoic acid synthesis is essential for early mouse post-implantation development. *Nat. Genet.* **21**, 444–448 (1999). 10192400 10.1038/7788

[80] Ribes, V., Le Roux, I., Rhinn, M., Schuhbaur, B. & Dollé, P. Early mouse caudal development relies on crosstalk between retinoic acid, Shh and Fgf signalling pathways. *Development* **136**, 665–676 (2009). 19168680 10.1242/dev.016204

[81] Pierani, A., Brenner-Morton, S., Chiang, C. & Jessell, T. M. A Sonic Hedgehog-independent, retinoid-activated pathway of neurogenesis in the ventral spinal cord. *Cell* **97**, 903–915 (1999). 10399918 10.1016/s0092-8674(00)80802-8

[82] Balaskas, N. et al. Gene regulatory logic for reading the sonic hedgehog signaling gradient in the vertebrate neural tube. *Cell* **148**, 273–284 (2012). 22265416 10.1016/j.cell.2011.10.047 PMC3267043

[83] Cohen, M., Briscoe, J. & Blassberg, R. Morphogen interpretation: the transcriptional logic of neural tube patterning. *Curr. Opin. Genet. Dev.* **23**, 423–428 (2013). 23725799 10.1016/j.gde.2013.04.003

[84] Lowery, L. A. & Sive, H. Strategies of vertebrate neurulation and a re-evaluation of teleost neural tube formation. *Mech. Dev.* **121**, 1189–1197 (2004). 15327780 10.1016/j.mod.2004.04.022

[85] Ordahl, C. P. & Douarin, N. M. L. Two myogenic lineages within the developing somite. *Development* **114**, 339–353 (1992). 1591996 10.1242/dev.114.2.339

[86] Lovell-Badge, R. et al. ISSCR guidelines for stem cell research and clinical translation: the 2021 update. *Stem Cell Rep.* **16**, 1398–1408 (2021). 10.1016/j.stemcr.2021.05.012 PMC8190668 34048692

[87] Roberts, B. et al. Systematic gene tagging using CRISPR/Cas9 in human stem cells to illuminate cell organization. *Mol. Biol. Cell* **28**, 2854–2874 (2017). 28814507 10.1091/mbc.E17-03-0209 PMC5638588

[88] Fan, Y. et al. hPSC-derived sacral neural crest enables rescue in a severe model of Hirschsprung’s disease. *Cell Stem Cell* **30**, 264–282 (2023). 36868194 10.1016/j.stem.2023.02.003 PMC10034921

[89] Berg, S. et al. ilastik: interactive machine learning for (bio)image analysis. *Nat. Methods* **16**, 1226–1232 (2019). 31570887 10.1038/s41592-019-0582-9

[90] Stirling, D. R. et al. CellProfiler 4: improvements in speed, utility and usability. *BMC Bioinform.* **22**, 433 (2021). 10.1186/s12859-021-04344-9 PMC8431850 34507520

[91] Schneider, C. A., Rasband, W. S. & Eliceiri, K. W. NIH Image to ImageJ: 25 years of image analysis. *Nat. Methods* **9**, 671–675 (2012). 22930834 10.1038/nmeth.2089 PMC5554542

[92] Schindelin, J. et al. Fiji: an open-source platform for biological-image analysis. *Nat. Methods* **9**, 676–682 (2012). 22743772 10.1038/nmeth.2019 PMC3855844

[93] Hao, Y. et al. Integrated analysis of multimodal single-cell data. *Cell* **184**, 3573–3587 (2021). 34062119 10.1016/j.cell.2021.04.048 PMC8238499

[94] McGinnis, C. S., Murrow, L. M. & Gartner, Z. J. DoubletFinder: doublet detection in single-cell RNA sequencing data using artificial nearest neighbors. *Cell Syst.* **8**, 329–337 (2019). 30954475 10.1016/j.cels.2019.03.003 PMC6853612

[95] Trapnell, C. et al. The dynamics and regulators of cell fate decisions are revealed by pseudotemporal ordering of single cells. *Nat. Biotechnol.* **32**, 381–386 (2014). 24658644 10.1038/nbt.2859 PMC4122333

[96] Van den Berge, K. et al. Trajectory-based differential expression analysis for single-cell sequencing data. *Nat. Commun.* **11**, 1201 (2020). 32139671 10.1038/s41467-020-14766-3 PMC7058077

[97] Gu, Z., Eils, R. & Schlesner, M. Complex heatmaps reveal patterns and correlations in multidimensional genomic data. *Bioinforma.* **32**, 2847–2849 (2016). 10.1093/bioinformatics/btw313 27207943

