# Supplementary material for: Modelling co-development between the somites and neural tube in human trunk-like structures
Source: Nat Cell Biol. 2025 Dec 16;27(12):2049–62. doi: 10.1038/s41556-025-01813-8 (PMC12717004; doi:10.1038/s41556-025-01813-8)
Supplement: Supplementary file 1 — Supplementary Fig. 1 and Table legends 1–3. [file 41556_2025_1813_MOESM1_ESM.pdf]

nature cell biology

Supplementary information https://doi.org/10.1038/s41556-025-01813-8

Modelling co-development between the somites and neural tube in human trunk-like structures

In the format provided by the authors and unedited

Supplementary Information Table of Contents:

Supplementary Figure ………..………………………………………………… 2

Supplementary Table Legends ………..…………..……………………………… 4

Supplementary Figures

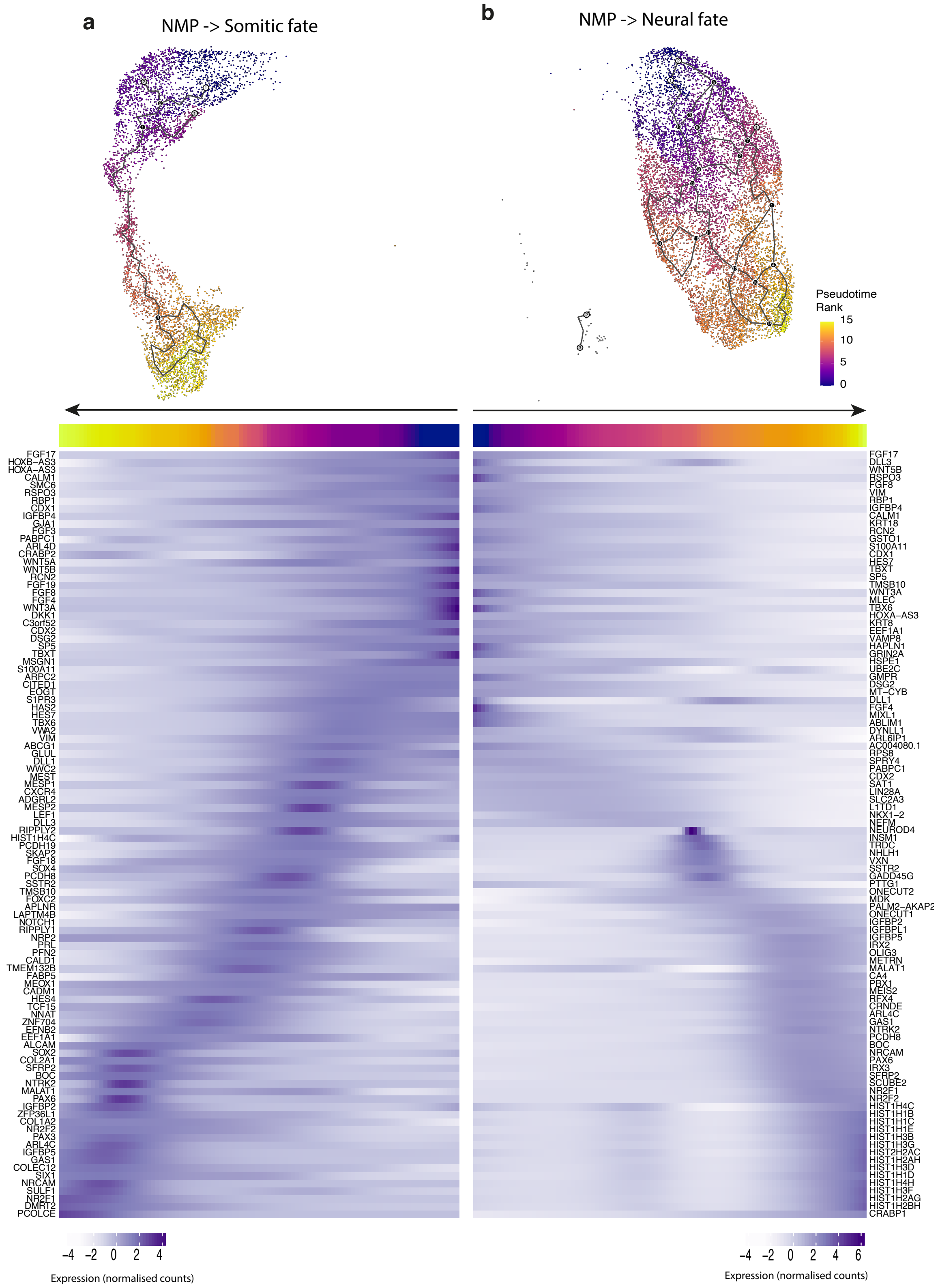

Supplementary Figure 1: Neural and mesodermal trajectory gene expression signatures. Monocle trajectory analysis showing two lineages from neuromesodermal progenitor (NMP) population towards somitic **(a)** and neural **(b)** populations (top), alongside ordering of cells by pseudotime with heatmap representation of the top 100 variable genes for each differentiation route (bottom). NMPs, Neuromesodermal progenitors.

Supplementary Table Legends

Supplementary Table 1: Chiron concentrations at pre-treatment and aggregation by cell line.

Supplementary Table 2: HCR probe list.

Supplementary Table 3: Light microscopy reporting summary
