# Supplementary material for: Modelling co-development between the somites and neural tube in human trunk-like structures
Source: Nat Cell Biol. 2025 Dec 16;27(12):2049–62. doi: 10.1038/s41556-025-01813-8 (PMC12717004; doi:10.1038/s41556-025-01813-8)
Supplement: Supplementary file 2 — Reporting Summary [file 41556_2025_1813_MOESM2_ESM.pdf]

nature portfolio

Corresponding author(s): Naomi Moris

Last updated by author(s): Oct 7, 2025

Reporting Summary

Nature Portfolio wishes to improve the reproducibility of the work that we publish. This form provides structure for consistency and transparency in reporting. For further information on Nature Portfolio policies, see our Editorial Policies and the Editorial Policy Checklist.

Statistics

For all statistical analyses, confirm that the following items are present in the figure legend, table legend, main text, or Methods section.

n/a	Confirmed	
☐	☒	The exact sample size (*n*) for each experimental group/condition, given as a discrete number and unit of measurement
☒	☐	A statement on whether measurements were taken from distinct samples or whether the same sample was measured repeatedly
☐	☒	The statistical test(s) used AND whether they are one- or two-sided<br>*Only common tests should be described solely by name; describe more complex techniques in the Methods section.*
☒	☐	A description of all covariates tested
☒	☐	A description of any assumptions or corrections, such as tests of normality and adjustment for multiple comparisons
☐	☒	A full description of the statistical parameters including central tendency (e.g. means) or other basic estimates (e.g. regression coefficient) AND variation (e.g. standard deviation) or associated estimates of uncertainty (e.g. confidence intervals)
☐	☒	For null hypothesis testing, the test statistic (e.g. *F*, *t*, *r*) with confidence intervals, effect sizes, degrees of freedom and *P* value noted<br>*Give P values as exact values whenever suitable.*
☒	☐	For Bayesian analysis, information on the choice of priors and Markov chain Monte Carlo settings
☒	☐	For hierarchical and complex designs, identification of the appropriate level for tests and full reporting of outcomes
☒	☐	Estimates of effect sizes (e.g. Cohen's *d*, Pearson's *r*), indicating how they were calculated

Our web collection on statistics for biologists contains articles on many of the points above.

Software and code

Policy information about availability of computer code

Data collection	MetaXpress (version 6.7.1.157), Zeiss ZEN Black 2.3 SP1, Imspector (7.5.4).

Data analysis

FIJI (version 2.14.0/1.54f)
Imaris (version 10.1.1)
Ilastik (version 1.4.0)
Cell Profiler (version 4.2.8)
Excel (version 16.92)
R (version 4.1.0)
CellRanger (version 6.0.1)
Seurat (version 4.4.0)
IntrinsicDimension (version 1.2.0)
Velocyto (version 0.17.8)
Python (version 3.6.4)
scVelo (version 0.2.4)
loompy (version 3.0.6)
scanpy (version 1.8.2)
Monocle3 (version 1.3.0)
TradeSeq (version 1.7.07)
ComplexHeatmap (version 2.2.0)
CellChat (version 2.1.2)

For manuscripts utilizing custom algorithms or software that are central to the research but not yet described in published literature, software must be made available to editors and reviewers. We strongly encourage code deposition in a community repository (e.g. GitHub). See the Nature Portfolio guidelines for submitting code & software for further information.

Data

Policy information about availability of data

All manuscripts must include a data availability statement. This statement should provide the following information, where applicable:

- Accession codes, unique identifiers, or web links for publicly available datasets
- A description of any restrictions on data availability
- For clinical datasets or third party data, please ensure that the statement adheres to our policy

The sequencing datasets generated during the current study are available in the GEO repository, GSE268451 with accession codes GSM8291523-5.

Research involving human participants, their data, or biological material

Policy information about studies with human participants or human data. See also policy information about sex, gender (identity/presentation), and sexual orientation and race, ethnicity and racism.

Reporting on sex and gender	NA
Reporting on race, ethnicity, or other socially relevant groupings	NA
Population characteristics	NA
Recruitment	NA
Ethics oversight	NA

Note that full information on the approval of the study protocol must also be provided in the manuscript.

Field-specific reporting

Please select the one below that is the best fit for your research. If you are not sure, read the appropriate sections before making your selection.

☒ Life sciences ☐ Behavioural & social sciences ☐ Ecological, evolutionary & environmental sciences

For a reference copy of the document with all sections, see nature.com/documents/nr-reporting-summary-flat.pdf

Life sciences study design

All studies must disclose on these points even when the disclosure is negative.

Sample size	No sample size calculations were performed. Sample sizes were determined based on the variability of the parameter under consideration and resource allocation. The chosen sample sizes are consistent with similar studies and preference was given to collecting biologically replicate data over further technical replicates.
Data exclusions	No data were excluded from the dataset, except for quantification of morphometrics where images of empty wells, those with debris, where

Data exclusions	structures were partially out of the field of view, where light intensity fell below a pixel threshold, or where manual checking identified poor segmentation. Bioinformatically, low quality cells or doublets (identified by DoubletFinder with theoretical rate of 7.5%) were removed from downstream analysis and cells were only kept if they met the following criteria: the mitochondrial content was within three standard deviations from the median, more than 500 genes were detected and more than 1,000 RNA molecules were detected.
Replication	Experiments were repeated several times (N), and often by independent researchers. All attempts at replication were successful; none of the findings shown could not be replicated. Records were kept of experimental outcomes and any attempts at replication that were aborted for technical reasons (e.g. failure to meet key checkpoints in the protocol).
Randomization	No randomization was involved in the study design, because hTLS structures were assumed to be independent.
Blinding	Investigators were not blinded to experimental conditions during experimental design, data collection or analysis. Blinding was neither practical nor relevant in the development of the culture protocol. Blinding was not required for quantitative image analyses, since measurements were made in an unbiased way (e.g. computational object detection).

Reporting for specific materials, systems and methods

We require information from authors about some types of materials, experimental systems and methods used in many studies. Here, indicate whether each material, system or method listed is relevant to your study. If you are not sure if a list item applies to your research, read the appropriate section before selecting a response.

Materials & experimental systems

n/a	Involved in the study
☐	☒ Antibodies
☐	☒ Eukaryotic cell lines
☒	☐ Palaeontology and archaeology
☐	☒ Animals and other organisms
☒	☐ Clinical data
☒	☐ Dual use research of concern
☒	☐ Plants

Methods

n/a	Involved in the study
☒	☐ ChIP-seq
☐	☒ Flow cytometry
☒	☐ MRI-based neuroimaging

Antibodies

Antibodies used	1:200 rabbit anti-Brachyury (Abcam, ab209665); 1:500 mouse anti-MEOX1 (ThermoFisher, TA-804716); 1:200 goat anti-SOX2 (R&D Systems, AF2018-SP), 1:200 rat anti-SOX2 (Invitrogen, 14-9811-82), 1:400 rabbit anti-PAX6 (ThermoFisher, 42-6600), 1:500 Phalloidin CruzFluor 647 (Santa Cruz, sc-363797), 1:10 mouse anti-PAX7 (DSHB, PAX7) and 1:500 rabbit anti-ALDH1A2 (Sigma-Aldrich, HPA01002). All secondary antibodies were raised in Donkey and diluted 1:500, and included Alexa Fluor 488-, 594- and 647 conjugated antibodies (Jackson ImmunoResearch).
Validation	All antibodies were tested for significant signal-to-background noise by whole mount immunoflourescence confocal microscopy. Secondary antibody-only controls were used to confirm specificity of observed signal. All primary antibodies were validated for sensitivity and specificity by the manufacturers, are reactive to human proteins, and have been previously published.

Eukaryotic cell lines

Policy information about cell lines and Sex and Gender in Research

Cell line source(s)	Human iPSC lines include HES7::Achillies (Pourquie lab; male), SOX2::mEGFP (FCI-AICS-0074-026; Allen Cell Collection; male), ZO-1::mEGFP (FCI-AICS-0023; Allen Cell Collection; male), beta-catenin::mEGFP (FCI-AICS-0058 cl.67; Allen Cell Collection; male), LaminB1::mTagRFP-T (FCI-AICS-0034 cl.62; Allen Cell Collection; male), H2B::mEGFP (FCI-AICS-0061 cl.36; Allen Cell Collection; male) and GM25256 (GM25256; Allen Cell Collection; male); and the human ES cell lines RUES2-GLR (Brivanlou lab; female) and H9 SOX2::H2B-tdTomato/T::H2B-GFP dual reporter (H9-SBR, Zhou lab; male).
Authentication	Cell lines were routinely STR profiled and subjected to low pass sequencing for karyotype checking.
Mycoplasma contamination	All cell lines tested negative for Mycoplasma.
Commonly misidentified lines (See ICLAC register)	No commonly misidentified lines were included in this study (ICLAC register version 13).

Animals and other research organisms

Policy information about studies involving animals; ARRIVE guidelines recommended for reporting animal research, and Sex and Gender in Research

Laboratory animals	CD-1 (BRCD) mice

Wild animals	NA
Reporting on sex	Sex was not considered as part of embryo analysis.
Field-collected samples	NA
Ethics oversight	All experiments carried out on mice were conducted according to the UK Animal (Scientific Procedures) Act under license PP6551133.

Note that full information on the approval of the study protocol must also be provided in the manuscript.

Plants

Seed stocks	*Report on the source of all seed stocks or other plant material used. If applicable, state the seed stock centre and catalogue number. If plant specimens were collected from the field, describe the collection location, date and sampling procedures.*
Novel plant genotypes	*Describe the methods by which all novel plant genotypes were produced. This includes those generated by transgenic approaches, gene editing, chemical/radiation-based mutagenesis and hybridization. For transgenic lines, describe the transformation method, the number of independent lines analyzed and the generation upon which experiments were performed. For gene-edited lines, describe the editor used, the endogenous sequence targeted for editing, the targeting guide RNA sequence (if applicable) and how the editor was applied.*
Authentication	*Describe any authentication procedures for each seed stock used or novel genotype generated. Describe any experiments used to assess the effect of a mutation and, where applicable, how potential secondary effects (e.g. second site T-DNA insertions, mosiacism, off-target gene editing) were examined.*

Flow Cytometry

Plots

Confirm that:

☒ The axis labels state the marker and fluorochrome used (e.g. CD4-FITC).

☒ The axis scales are clearly visible. Include numbers along axes only for bottom left plot of group (a 'group' is an analysis of identical markers).

☒ All plots are contour plots with outliers or pseudocolor plots.

☒ A numerical value for number of cells or percentage (with statistics) is provided.

Methodology

Sample preparation	Pluripotent stem cell cultures were washed with PBS and incubated with Accutase for 4 minutes at 37 °C to obtain a single-cell suspension. Following enzymatic dissociation, cells were centrifuged and washed twice with PBS, then resuspended in fresh culture medium supplemented with 10 µM Y-27632 (ROCK inhibitor) to enhance cell survival. The cell suspension was kept on ice and transported to the flow cytometry facility for sorting.
Instrument	BD FACSAria™ Fusion flow cytometer
Software	BD FACSDiva™ software
Cell population abundance	The abundance of the mCherry-positive cell population ranged from approximately 0.1% to 2% of the total input cell population.
Gating strategy	Cells were initially gated based on size and granularity (SSC-A vs. FSC-A). Singlets were then identified using FSC-H vs. FSC-A gating. mCherry-positive cells were subsequently selected (gate P3) and sorted at one cell per well to derive monoclonal cell lines.

☒ Tick this box to confirm that a figure exemplifying the gating strategy is provided in the Supplementary Information.
